# Supraspinal commands have a modular organization that is behavioral context specific

**DOI:** 10.1016/j.cub.2025.07.066

**Published:** 2025-08-22

**Authors:** Joanna Y.N. Lau, James E. Fitzgerald, Isaac H. Bianco

**Affiliations:** 1 Department of Neuroscience, Physiology, & Pharmacology, UCL, London WC1E 6BT, UK; 2 Department of Neurobiology, Northwestern University, Evanston, IL 60208, USA; 3 Janelia Research Campus, Howard Hughes Medical Institute, Ashburn, VA 20147, USA; 4 NSF-Simons National Institute for Theory and Mathematics in Biology, Chicago, IL 60611, USA

## Abstract

Animals generate a range of locomotor patterns that subserve diverse behaviors, and in vertebrates, the required supraspinal commands derive from reticulospinal neurons in the brainstem. Yet how these commands are encoded across the reticulospinal population is unknown, making it unclear whether a universal control logic generates the full locomotor repertoire or if distinct sets of command modules might compose movement in different behavioral contexts. Here, we used calcium imaging, high-resolution behavior tracking, and statistical modeling to comprehensively survey reticulospinal activity and relate single-cell activity to movement kinematics as larval zebrafish generated a broad diversity of swim types. We found that reticulospinal population activity had a low-dimensional organization and identified 8 functional archetypes that provided a succinct and robust encoding of the full range of locomotor actions. Across much of locomotor space, 5 functional archetypes supported multiplexed control of swim speed and independent control of direction, whereas an independent set of 3 functional archetypes controlled the specialized swims that zebrafish use during hunting to orient toward prey. Overall, our study reveals a modular supraspinal control architecture that is partitioned according to behavioral context.

## Introduction

Locomotion is a fundamental and universal part of the motor repertoire of animals and typically comprises various modes of movement that subserve a broad range of behaviors including exploration, social interactions, escape, and hunting. Evidence suggests that an evolutionarily conserved neural architecture controls locomotion across vertebrate species.^1–3^ In this blue-print, executive circuits in the spinal cord function as central pattern generators to produce rhythmic and coordinated patterns of muscle activations and receive descending commands from supraspinal pathways that function to initiate, modulate, and terminate movement. The principal source of these supraspinal commands are the reticulospinal neurons (RSNs), a heterogeneous set of cells that extend from the caudal midbrain through the pontomedullary hindbrain and act as an interface that, on the one hand, integrates diverse upstream inputs and, on the other, projects large, fast-conducting axons to innervate spinal targets.^4,5^

Recent studies in lamprey, zebrafish, and mice have been successful in linking specific locomotor functions to defined sub-groups of RSNs.^2,6^ For example, a combination of cellular-resolution imaging, electrophysiology, and pharmacology led to the discovery of RSN subpopulations in lamprey whose activity is compatible with initiating, maintaining, and halting locomotion.^7^ In mice, genetic and circuit tracing tools have allowed subsets of RSNs defined by anatomical locus, neurotransmitter expression, and projection targets to be linked to control of speed, direction, and locomotor arrest.^8–11^ Larval zebrafish have a rich and well-characterized locomotor repertoire^12,13^ and are an appealing model vertebrate for studying locomotor control. At larval stages, the reticulospinal population is especially compact and is both genetically and optically accessible,^6^ and hindbrain neurons conform to a regular organization according to gene expression, birthdate, and morphological and physiological properties.^14–17^ Pioneering studies provided evidence for a population code mediated by the Mauthner cell and its segmental homologs, which controls a specific locomotor behavior, the C-start escape.^18,19^ Subsequent studies have implicated specific RSNs in the control of speed,^20,21^ tail posture,^22^ directional control,^23–25^ and avoidance swims to visual,^26^ nociceptive,^27^ and homeostatic^28^ threats.

However, it is still unclear how the reticulospinal population functions as a whole to control locomotor diversity. Specifically, although much evidence points to a modular organization in which subgroups of RSNs control component features of the overall locomotor output, it is not clear how kinematic variables such as speed and direction are encoded across the entire population of descending neurons. Moreover, animals express different forms of locomotion in various contexts, and it is not known whether supraspinal control has a universal architecture that functions similarly across behavioral space, or whether there might be subsets of neurons, or spatiotemporal patterns of activity, that play more specialized roles under specific behavioral contexts. Previous studies have typically been unable to address these issues by virtue of having examined a limited part of the locomotor repertoire and/or restricting focus to a limited subset of RSNs. Resolving these questions therefore requires functional assessment of the majority of the reticulospinal population, at single-cell resolution, in the context of a broad range of naturalistic locomotor outputs.

In this study, we used calcium imaging to comprehensively monitor RSN activity at cellular resolution while larval zebrafish generated a broad diversity of swim types that were evoked in a context-dependent manner by sensory cues. Across a large part of locomotor space, the RSN population showed broadly distributed activity that changed in a continuous, graded manner, in alignment with motor kinematic variation. However, by modeling the responses of single neurons, we found that this activity had a low-dimensional organization that was well-matched to the complexity of behavior and could be summarized by 8 “functional archetypes,” each encompassing a range of anatomically defined cell types. The combinatorial activity of 5 archetypes appeared to control the majority of swims, with multiplexed encoding of swim speed and independent encoding of direction. Remarkably, an independent set of 3 archetypes were associated with the specialized swims that larvae deploy during hunting. Overall, our study uncovers an organizational logic within the RSN command population in which modules encoding speed and direction act combinatorially to compose behavior but with additional context-specific stratification.

## Results

### Calcium imaging of reticulospinal activity during behavior

**Figure 1:**
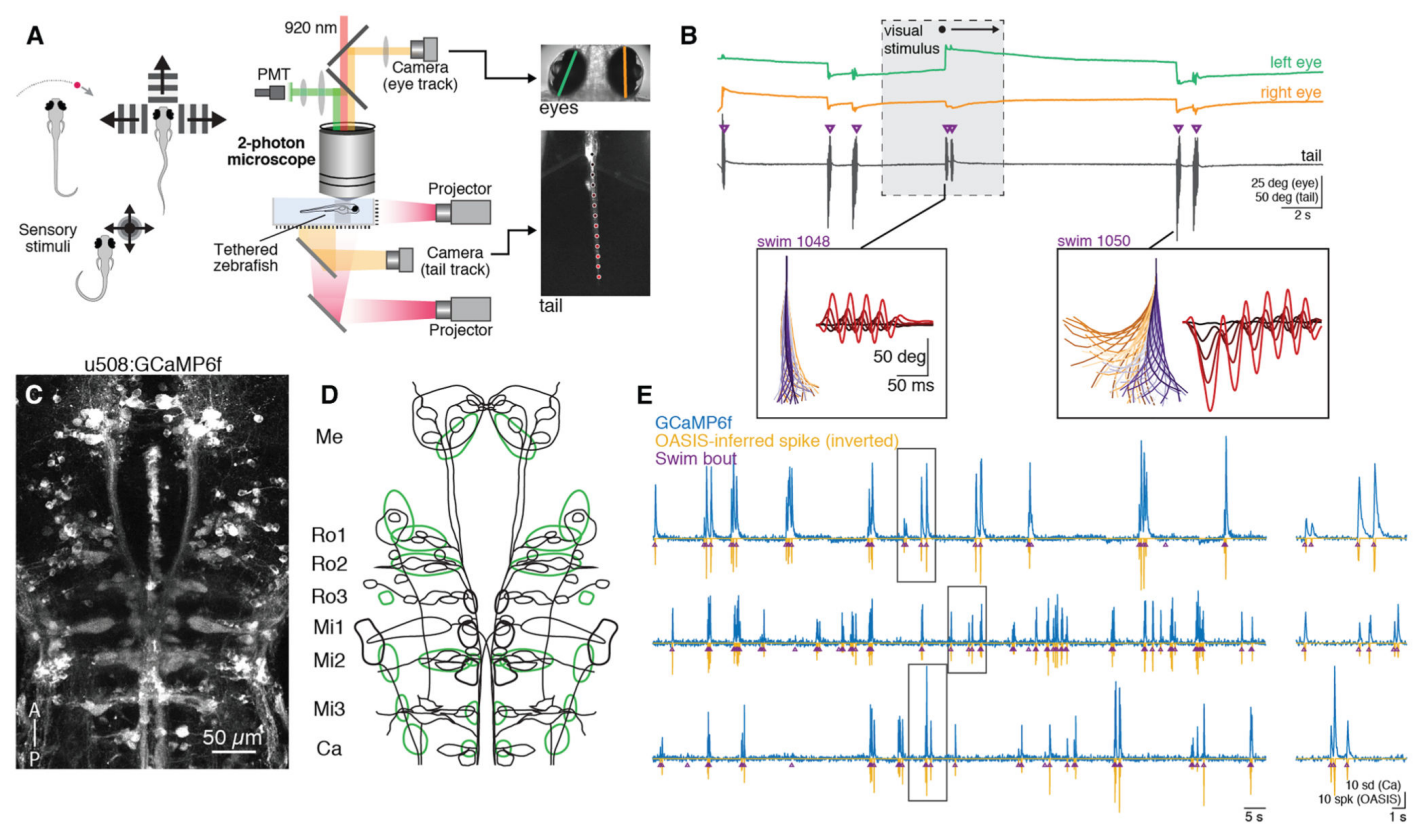
Calcium imaging of RSNs during behavior (A) Schematic of 2-photon calcium imaging and concurrent behavioral tracking. A subset of the sensory stimuli are illustrated and insets show eye and tail tracking. (B) Behavioral data for an example 30 s epoch during which a prey-like moving spot was presented (shaded box). Bottom images detail 2 swim bouts showing tail skeletons (time runs orange → purple) and time-varying cumulative tail bend angle (black → red corresponds to curvature along progressively more distal lengths of the tail). Swim 1048 was coincident with a convergent saccade as the animal initiated a hunting response toward the prey-like stimulus. (C) u508:GCaMP6f expression in the mid/hindbrain. Maximum intensity projection of image volume. A, anterior; P, posterior. (D) Schematic of RSN array (full labeling in Figure S1). (E) Calcium time series (blue) and OASIS-inferred spiking process (yellow, inverted for visualization clarity) for 3 example neurons. Boxed periods shown at expanded time scale on right. Purple triangles indicate times of swim bouts. See also Figure S1.

To comprehensively survey RSN activity, we combined cellular-resolution 2-photon calcium imaging with behavioral tracking in larval zebrafish (6–7 days post-fertilization, dpf) (Figure 1A). At larval stages, locomotion is naturally segmented into short periods of swimming (bouts) separated by brief quiescent periods (interbout intervals), and bouts with distinct kinematic properties are generated in different behavioral contexts.^12,13^ To elicit a variety of bout types in tethered animals, we therefore presented a range of sensory cues including small prey-like moving spots to evoke hunting-related J-turns,^29,30^ drifting gratings to evoke optomotor forward swims and turns,^23,31^ and looming disks and water puffs to trigger fast avoidance swims.^32,33^ We recorded spontaneous and stimulus-evoked behavior using high-speed cameras to track eye (60 Hz) and tail (420 Hz) motion (Figure 1B).

We took advantage of the KalTA4u508 transgene^34^ to drive expression of GCaMP6f in RSNs (Figures 1C, 1D, and S1). Prior studies have visualized the canonical population of RSNs by applying tracers to their descending axons in the spinal cord.^14,35–37^ We therefore assessed the extent of RSN labeling in *u508*:GCaMP6f animals by comparing transgene expression with spinal backfill labeling (Figure S1A). Of the 72 reticulospinal cell types that have been described in larval zebrafish, 66 were reliably labeled in the transgenic line (*n* = 19 animals; Figure S1C). We observed a high labeling probability for most “singly occurring” types (e.g., MeLc and Mauthner cell), where one identified neuron exists in each hemisphere, as well as multiple transgenically labeled neurons per animal for most “multiply occurring” types (e.g., RoV3 and MiV2 clusters). We also identified a singly occurring neuron in the Ro2 segment that has not previously been described and named it RoM2r following convention^36^ (Figure S1D). KalTA4u508 additionally drove GCaMP expression in several clusters of cells in the tegmentum that are not labeled by spinal backfill (Figures S1B and S1C). These might include spinally projecting neurons that are not readily labeled by retrograde tracing^17^ and were given labels describing their location relative to canonical RSNs (“other *u508* cells,” green labels in Figures 1D and S1B).

We performed calcium imaging of focal planes spanning the portion of the mid/hindbrain tegmentum that contains the reticulospinal population (~16 planes/animal at 5 μm *z*-spacing), manually segmented individual somata (18,750 cells from 67 animals), and assigned anatomical labels as per Figure S1B. Next, we applied the OASIS^38^ deconvolution algorithm to account for indicator dynamics and better localize neuronal activity to swim bouts (Figure 1E). All further analyses used these OASIS-inferred “spikes” (spk), although we note that this activity descriptor is not expected to equate to action potential counts or firing rates. In accordance with previous observations,^27,39,40^ activity traces showed clear coincidence of RSN activity with swim bouts, with minimal activity during intervening rest periods (Figure 1E).

### Zebrafish larvae generate diverse swim bouts that form a kinematic sequence

Studies in larval zebrafish have described both continuous variation in swim kinematics as well as distinct bout “types” that are deployed in a context-dependent manner.^12,13,26,33,41–46^ We therefore characterized the detailed kinematics and diversity of swims evoked in tethered animals under our experimental conditions.

**Figure 2:**
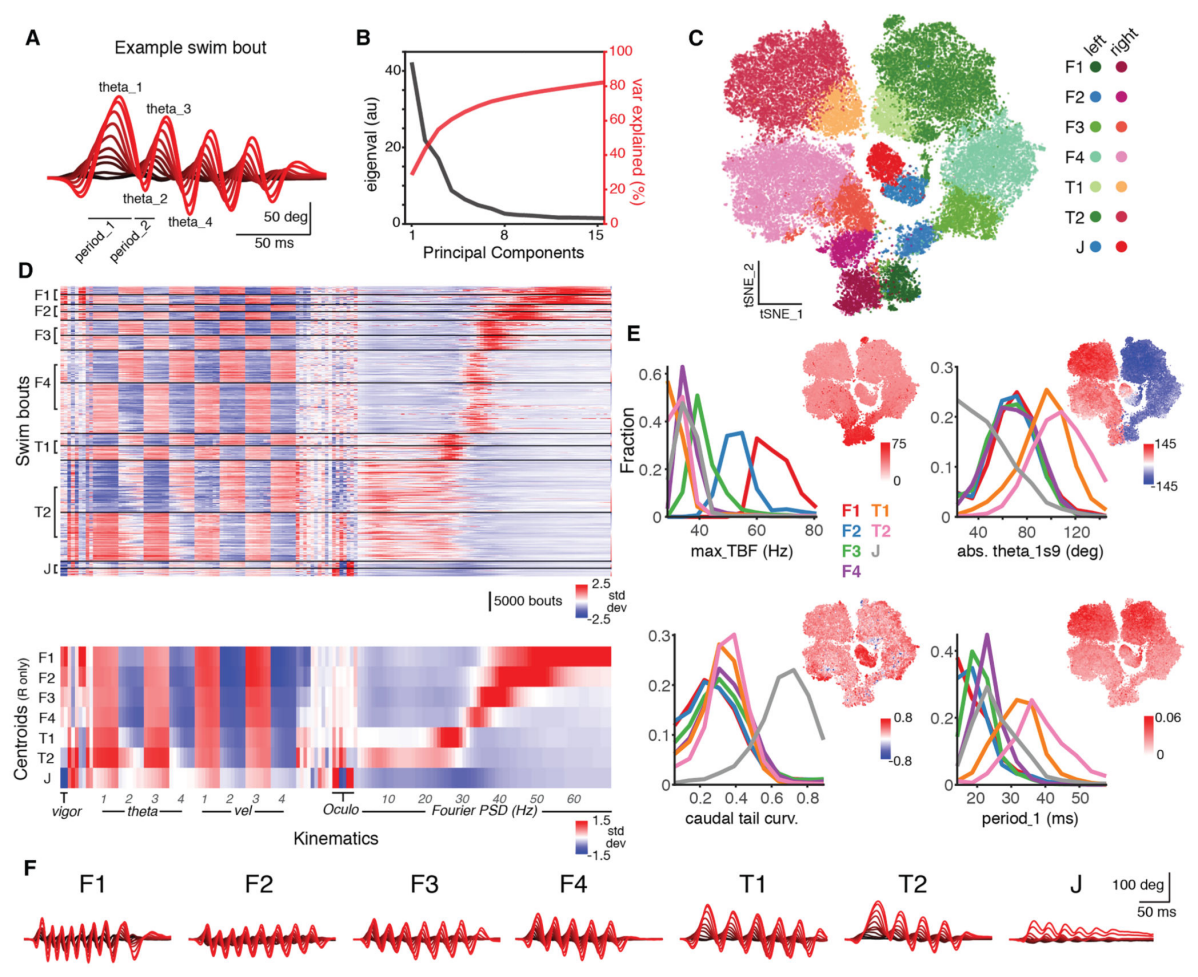
Swim bouts form a kinematic sequence (A) Example swim bout, shown as time-varying cumulative bend angle along the length of the trunk/tail (rostral → caudal indicated black → red). Some example kinematics are illustrated. *theta_1–4*, bend angle for half-beats 1–4; *period_1,2*, duration of half-beats 1 and 2. (B) Principal-component eigenvalues of the kinematic covariance matrix and cumulative percentage of variance explained by the principal components. (C) t-SNE embedding of swim bouts, colored by bout label (determined by an independent clustering procedure, see Figure S2). (D) *Top*: Kinematic vectors of all swim bouts, grouped by type and further subdivided into left- (upper rows) and right-lateralized (lower rows) swims. *Bottom*: Kinematic centroids for 7 (right-lateralized) bout types. Each kinematic feature (column) was normalized (z-scored) within fish. Kinematics related to swim vigor, bend angles (theta), and velocities (vel) over four half-beats, oculomotor parameters, and Fourier coefficients are indicated. For detailed breakdown see Table S1. (E) Distributions of several kinematic features across 7 bout types (left and right swims combined). Insets show t-SNE embedding, colored by kinematic value. Clockwise from top left: *max_TBF*, maximum tail-beat frequency, which separates different types of forward swim; *theta_1s9*, peak bend angle at tail segment 9 during half-beat 1, which is greater for T1 and T2 turns; *period_1*, duration of half-beat 1, which is longer for turns; *caudal tail curve* (fcC1), fraction of total bend angle localized to caudal tail segments during half-beat 1, which is elevated for J-turns. (F) Example (rightward) bouts of each type. See also Figure S2 and Table S1.

Locomotor space showed continuous variation in motor kinematics and was approximately eight-dimensional. To show this, we segmented individual swim bouts from tracking data and extracted 152 kinematic features characterizing each (141,831 bouts from 67 animals; STAR Methods). These included measures of swim vigor and lateralization, bend amplitudes and angular velocities for the first four half-beats of each swim, changes in eye position, and Fourier coefficients quantifying power across a range of frequencies (Figure 2A and full list in Table S1). We estimated the dimensionality of behavioral space from the eigenvalues of the kinematic covariance matrix, which revealed that swims predominantly varied along 8 kinematic axes (participation ratio = 7.94; 8 PCs explain 73% variance; Figure 2B). By embedding bouts in a two-dimensional space (principal-component analysis [PCA] followed by t-distributed stochastic neighbor embedding [t-SNE]), we observed that several kinematics changed systematically across the embedding space (Figures 2E and S2D). Features related to locomotor direction tended to vary along t-SNE dimension 1 (e.g., theta_1s9, describing bend angle of first half-beat), while features related to speed showed smooth variation aligned with t-SNE dimension 2 (e.g., tail-beat frequency, TBF).

To provide a convenient heuristic to describe locomotor space (and associated neural activity), we defined a small number of categorical bout types, which formed a ‘kinematic sequence.’ Briefly, we developed a pipeline based on hierarchical agglomerative clustering to identify groups of bouts with correlated kinematic profiles (Figures S2A–S2C; STAR Methods). This process identified 7 bout types, with each having left- and right-lateralized variants (14 labels in total; examples in Figure 2F). Bouts of each type formed coherent groups that tiled the embedding space in a sequence that was symmetrically repeated for left- and right-lateralized swims (Figure 2C). This kinematic sequence was also evident from the dendrogram of correlation distances between bout type centroids (Figure S2C) and orderly changes in the values of multiple kinematics when feature vectors were organized by bout type (Figure 2D). Specifically, swim vigor, TBF, and angular velocities progressively decreased across the kinematic sequence F1 → F2 → F3 → F4 → T1 → T2 → J, whereas half-beat amplitudes increased, reaching their greatest values with T1 and T2 bouts (Figures 2D, 2E, and S2D).

The swim bouts generated by tethered animals encompassed the large majority of the cycle frequency range previously described for freely swimming larval zebrafish (~20–80 Hz^13,20,41,47^), as well as characteristic features of routine turns and hunting-related J-turns. Thus, F1 swims had maximum tail-beat frequencies exceeding 60 Hz (Figure 2E) (upper peak in PSD spectrum centered at 56 Hz; Figure S2E), and peak cycle frequencies progressively decreased for F2 (upper PSD peak 43 Hz), F3 (36 Hz), and F4 (32 Hz) bout types. T1 and T2 bouts resembled the routine turns of freely swimming larvae, with elevated power at low frequencies (~4 Hz, describing the slow amplitude envelope of these swims; Figures 2D and S2E) and large amplitude and long duration of the first and third half-beats (Figures 2D and 2E), in accordance with Huang et al.^24^ These turns were differentiated by a switch in laterality of the second half-beat relative to the first, from contraversive (T1) to ipsiversive (T2) (Figure 2D and theta_2s6 in Figure S2D), which likely corresponds to increased turn strength for T2. Finally, J-turns comprised a markedly distinct bout type (Figure S2C), characterized by low swim vigor (Figure 2D) and bending concentrated toward caudal tail segments (Figure 2E), features that are believed to enable precise reorientation toward prey targets with minimal hydrodynamic disturbance.^43^ In accordance with their deployment at the initiation of hunting, they were associated with a large increase in ocular vergence (Figure S2D).^29,48^

All types of swim bout were generated spontaneously, but different bout types were selected in different sensory contexts (Figure S2F). Leftward and rightward optomotor gratings almost exclusively evoked left- and right-directed T2 bouts, suggesting similarity to the routine turns of freely swimming larvae.^13,23^ By contrast, forward gratings produced a broader range of swims, including F3 and F4 types. Prey-like stimuli were the most effective in evoking J-turns^29,30^ and, in our classification, dark flashes evoked T2 turns. Water puffs to the (left) ear most frequently evoked fast (rightwards) F1 swims. Note that we only evoked escape-like swims (resembling C-starts) at low frequency under our experimental conditions, so Mauthner-dependent escapes^18,19,26,44^ are poorly represented in this dataset.

### Reticulospinal population activity shows smooth, systematic variation across locomotor space

**Figure 3:**
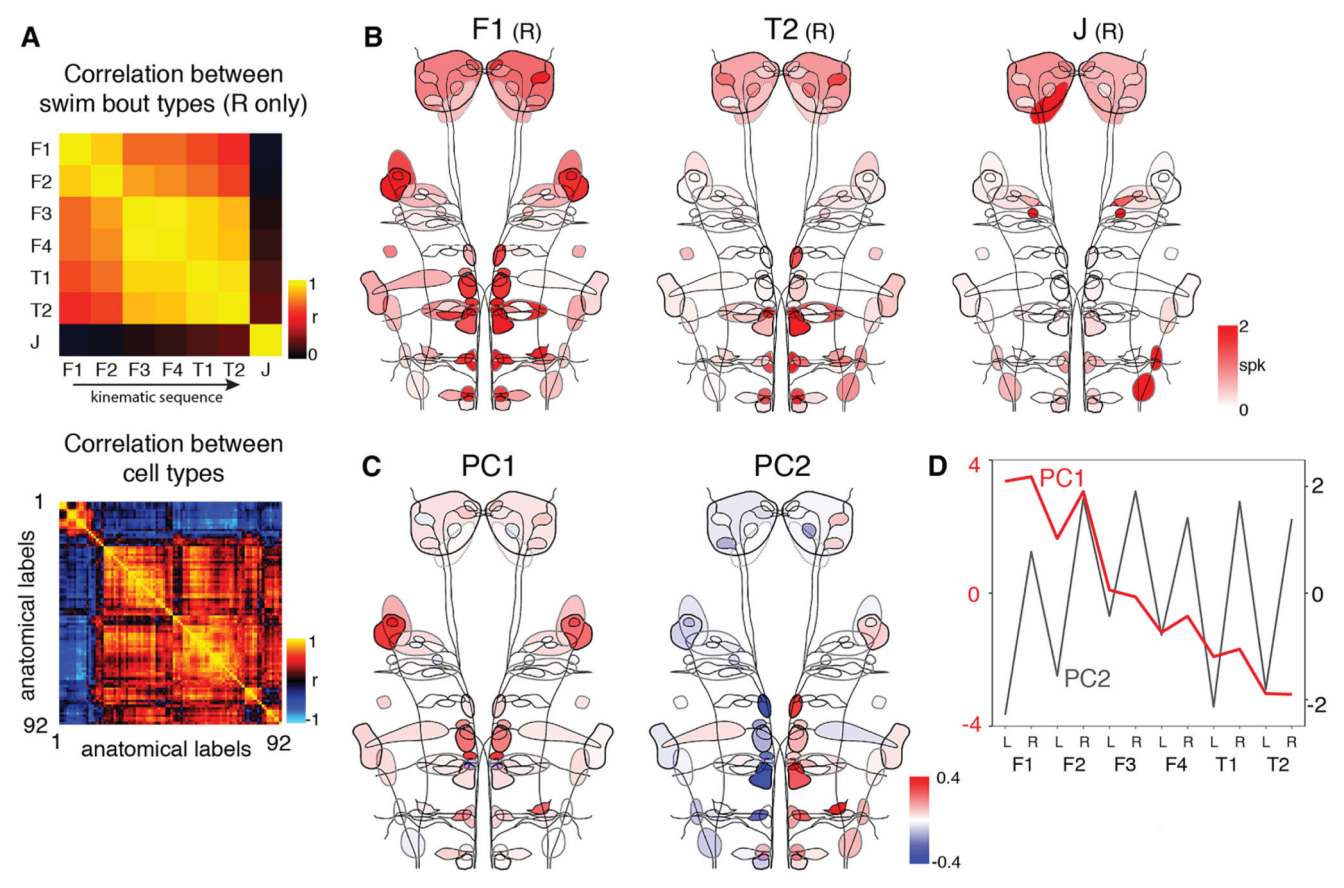
Reticulospinal population activity varies systematically across locomotor space (A) *Top*: Pearson’s correlation between bout type activity vectors (right-lateralized swims only). The input matrix was of the form cell types × bout types, such that each column vector quantified the mean spike count across cells belonging to each anatomical type. *Bottom*: Pearson’s correlation between cell type activity vectors (input matrix was bout types × cell types). Activity for all swims (left and right) was used, and cell types have been ordered according to correlation distance between activity vectors. (B) RSN recruitment for 3 bout types, representing the extremes of the kinematic sequence (right-lateralized F1, T2, and J-turns). Maps show mean spike counts across all recorded cells. (C and D) Principal-component analysis was used to identify the main axes of variation in neural activity across bout types (excluding J-turns). (C) Loadings for first two PCs. (D) The first two PCs vary across bout types. See also Figures S3 and S4.

We next examined how this motor diversity relates to RSN activity. To obtain an overview of reticulospinal population activity, we first examined patterns of neuronal recruitment associated with the 7 bout types that span locomotor space (Figures 3, S3, and S4). Notably, RSN activity was specific to swim events, as recruitment maps for “mock” bouts (randomly sampled from periods when the fish was not swimming) showed minimal activity (Figure S4B).

RSNs showed broad recruitment for most bout types, with a smooth progression in the pattern of supraspinal activity that paralleled changes in motor kinematics. Specifically, recruitment maps displayed gradual changes in activity along the kinematic sequence F1 → T2 (Figures 3B and S3), and population activity vectors were highly correlated (Figure 3A). In particular, bout types that were adjacent in kinematic space showed the largest correlation coefficients. To characterize this variation, we performed PCA on the population activity matrix (excluding J-turns; Figures S4C and S4D). The first principal component (which accounted for 43.8% variance) declined along the sequence F1 → T2 (Figure 3D), in parallel to the progressive decrease in swim vigor and TBF. This PC had anatomically symmetric positive loadings onto several cell types including RoR1, RoL1, MiV1, MiR1, MiR2, MiT, and neurons of the nucleus of the medial longitudinal fasciculus (nMLF) (Figure 3C), suggesting this broad set of neurons might be associated with aspects of speed control.

We also observed lateralized RSN activity, which increased along the kinematic sequence F1 → T2. The direction of asymmetry was reflected in PC2 (21.5% variance), which alternated in sign between left and right swims (Figure 3D). We further analyzed lateralization by comparing the activity of each cell type in the right versus left hemisphere (Figure S3 bottom row). This revealed lateralized activity in RoV3, MiV1, and MiV2 clusters, ipsilateral to swim direction. Asymmetric activity was strongest for the highly lateralized T1 and T2 bouts, in agreement with previous observations during optomotor stimulation^23^ and fictive turns.^24^ Although most pronounced during turns, lateralization was also apparent, albeit to a lesser extent, for F1–4. This indicates that even for swims that are likely to produce forward locomotion with minimal turning, descending commands are nonetheless lateralized in correspondence with the side of the body initiating movement (i.e., the first half-beat of a swim).

J-turns displayed a unique pattern of RSN activity (Figures 3A, 3B, and S3). Compared with other swims, population activity was sparse and, surprisingly, there was minimal recruitment of the ventral RSNs (RoV3, MiV1, and MiV2) that are involved in other types of turn. Instead, J-turns associated with asymmetric activity in laterally located *u508* populations (“Mi3 lateral” and “Ca1 lateral”), ipsilateral to turn direction. A second striking feature was strong, symmetric activation of the newly described RoM2r cells.

Overall, many RSNs were engaged across a broad range of swims, and the magnitude and lateralization of population activity varied smoothly and systematically across much of locomotor space. This suggests that continuous variability in RSN activity may control continuous variation in locomotor kinematics.

### Generalized linear modeling of single-cell activity

**Figure 4:**
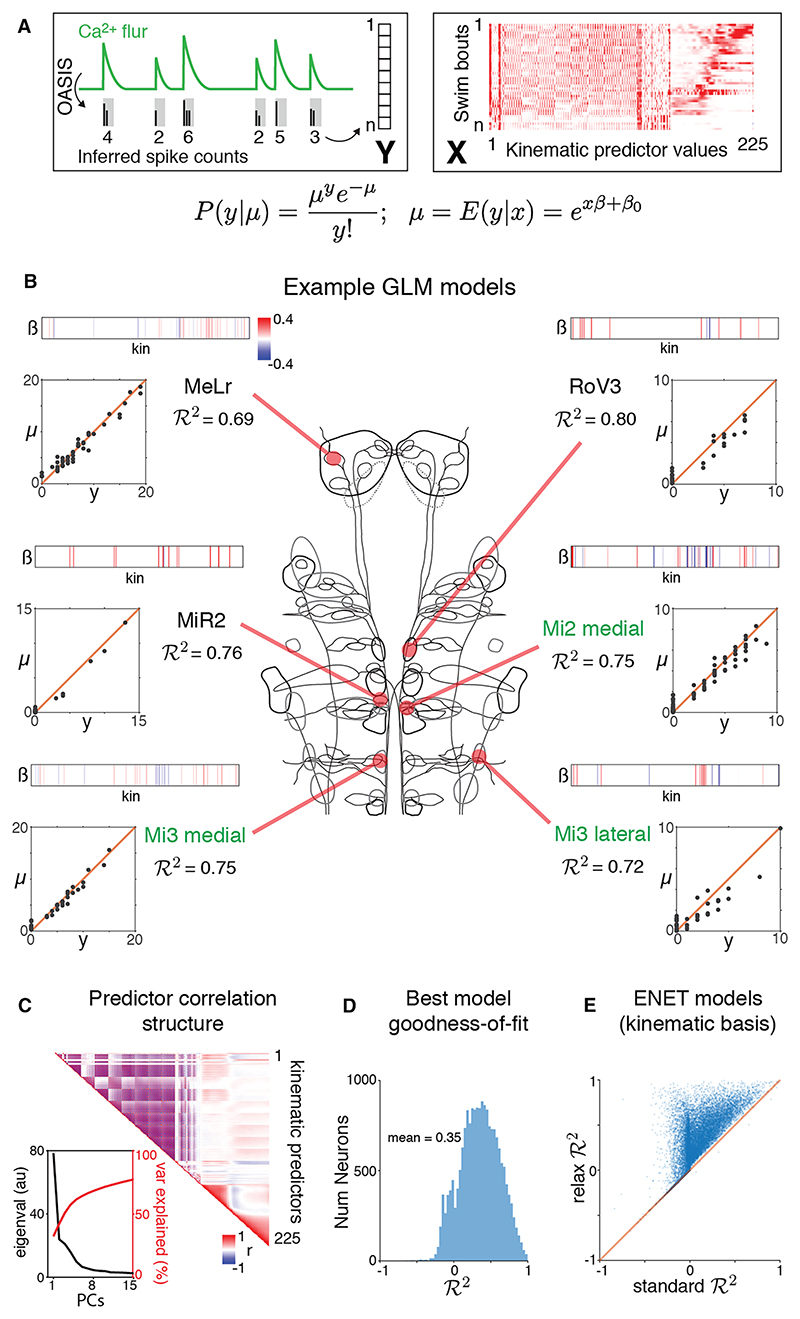
Single-neuron modeling (A) For each neuron, a spike count was inferred for each swim bout (vector *Y*, left) and kinematic predictor values were computed for the same *n* bouts (matrix *X*, right). Generalized linear regression was used to model expected spike counts, *μ*, as a function of motor kinematic predictors, *x*, assuming spikes follow a Poisson distribution. For further details see STAR Methods. (B) Example models. For each cell, we show the fitted model coefficients (*β*), goodness of fit (cross-validated fraction of deviance explained, *R*^2^), and OASIS-inferred spikes (*y*) versus the model prediction (*μ*). Red lines have a unity slope. Note that *β* is represented as a row vector here and in subsequent figures, for visualization. (C) Correlation matrix for all 225 kinematic predictors. Inset shows eigenvalues and cumulative variance explained by top principal components. (D) Distribution of best regression model *R*^2^, for all neurons. (E) Comparison of *R*^2^ (for models fitted in the basis of motor kinematics) either with or without relaxation. Relaxing model coefficients improves cross-validated performance. See also Table S1.

Recent work has revealed that neurons with diverse functional properties are intermingled even within limited subregions of the reticulospinal complex.^2,3,6^ Motivated by this, we developed a statistical modeling framework to quantitatively relate the activity of individual neurons to swim kinematics (Figure 4A). Specifically, we fitted generalized linear models (GLMs) that mimicked neuronal spiking variability by assuming that the number of OASIS-inferred spikes follows probabilistically from a Poisson distribution. To relate activity to behavior, we used the canonical link function for the Poisson GLM, which models each neuron’s “firing rate” as an exponentiated linear function of the motor kinematics. A large number of kinematics describe signed variation along the left-right axis. However, because neurons might encode movement in one direction,^24^ we converted these features to unsigned pairs of model predictors, split for left versus rightward movement (e.g., theta_1s9 becomes theta_1s9-L and theta_1s9-R; Table S1). Performance was quantified using the cross-validated fraction of deviance explained (*R*^2^), which generalizes the cross-validated *R*^2^ statistic to non-Gaussian noise models.

Building accurate and interpretable models of RSN activity requires statistical techniques that can effectively cope with many correlated behavioral predictors. To this end, we used elastic net regularization (ENET^49^) with 2 important adaptations (STAR Methods). First, we implemented a two-stage fitting procedure in which ENET modeling is followed by ridge regression to correct for known over-shrinkage of coefficients by ENET; this process is called “relaxation” in statistics.^50^ Second, we fitted models both in the basis of motor kinematics and additionally in the basis of their singular value decomposition (SVD) left-singular vectors (“modes”). In the latter case, model coefficients were mapped back into the kinematic basis for visualization and analysis. This procedure aided interpretability by “smoothing” coefficient values across correlated predictors.

We used this framework to model every neuron in our dataset (18,750 neurons from 67 fish). Figure 4B shows examples of model fits from different regions of the reticulospinal complex. Despite variability in indicator expression and the multicollinearity of behavioral predictors (Figure 4C), our procedure produced accurate models for many cells (Figure 4D). ENET outperformed simple ridge regression (data not shown), and our procedure to relax ENET coefficients improved cross-validated performance (Figure 4E). For most cells, the best fit was achieved in the basis of motor kinematics (84.9% of 18,750 cells), but a substantial minority were best fitted using kinematic modes (14.1%). We next used these GLM models to compare kinematic tuning across the entire dataset of RSNs.

### Reticulospinal activity can be summarized with eight functional archetypes

**Figure 5:**
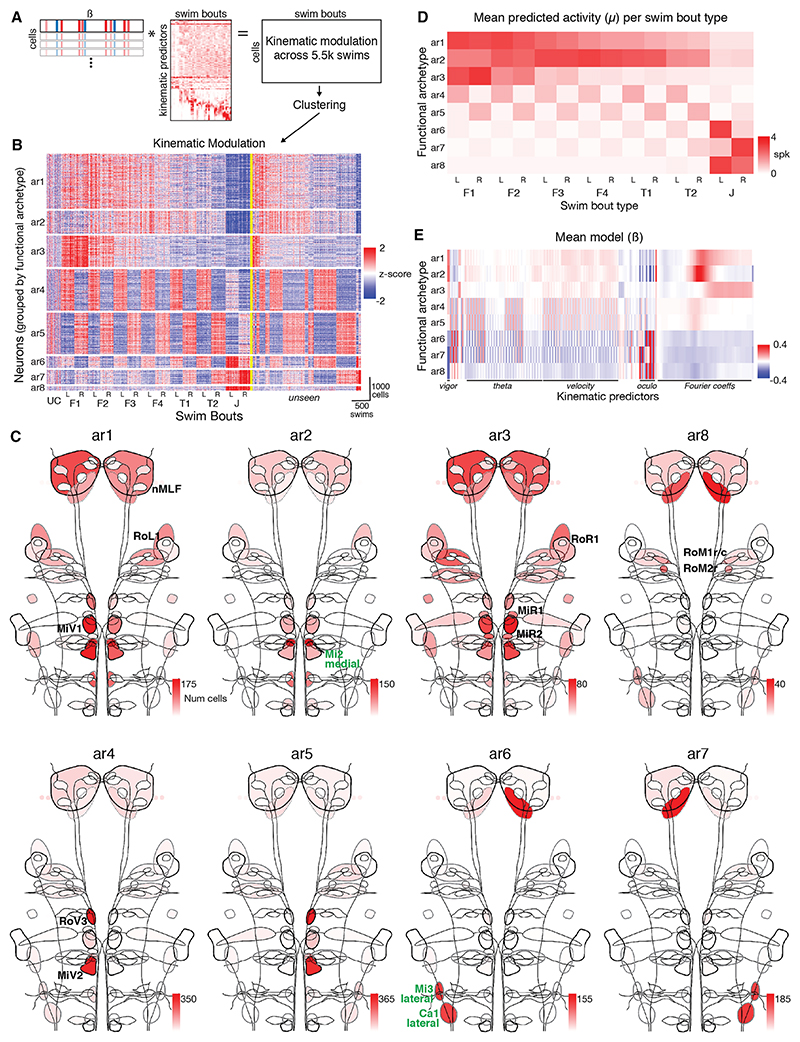
RSN activity can be described by 8 functional archetypes (A) Schematic showing estimation of “kinematic modulation” (*Xβ*) across a common set of ~5,000 swim bouts, using neurons’ best GLM models, followed by clustering. Note that *X* and *Xβ* are represented as their transposes to agree with our style convention of plotting *β* as a row vector. (B) Kinematic modulation vectors of all clustered neurons, organized by functional archetype (ar1–8). Similar patterns of modulation across swim bouts are observed within each archetype. Bouts to the right of the yellow line did not form part of the clustering process. Number of cells, *n* = 2,349 (ar1), 1,047 (ar2), 1,405 (ar3), 1,826 (ar4), 1,821 (ar5), 585 (ar6), 677 (ar7), and 231 (ar8). UC, unclassified swim bouts. (C) Maps showing number of cells of each anatomical label assigned to each functional archetype. (D) Model-predicted activity, *μ*, of each archetype across bout types. Values are means across cells belonging to each archetype. (E) Mean ENET model (*β*, fitted in the basis of singular vectors with relaxed coefficients) across cells assigned to each archetype. See also Figures S5–S7 and Table S1.

We used our single-cell GLM models to explore the diversity of functional properties across the reticulospinal population. It is challenging to directly compare vectors of model coefficients because ENET could select different predictors from a correlated set to explain the activity of functionally similar cells. Instead, we used the fitted models to predict how the activity of the full set of neurons would vary across a large and diverse set of swim bouts sampled from our dataset. Specifically, the best model for each neuron (highest *R*^2^) was used to calculate *Xβ* across a large, common set of swims (Figure 5A). We refer to this representation of predicted activity as “kinematic modulation,” because it defines how the kinematics of each bout change the cell’s baseline firing level, exp(*β*_0_) (Figure 4A). Although the kinematic modulation multiplicatively changes baseline firing rate with magnitude exp(*Xβ*), we emphasize its unexponentiated form throughout as this allows upward and downward modulation to contribute comparably to the activity representation. We estimated kinematic modulation across 5,573 randomly selected swims that represented the entirety of locomotor space from F1 bouts to J-turns (Figure 5B).

This analysis predicted that reticulospinal population activity has a low-dimensional organization. We showed this in several ways. First, we analyzed the eigenvalues of the kinematic modulation covariance matrix and estimated its dimensionality to be around 8 (participation ratio = 7.83; 8 PCs explain 75.9% of variance; Figure S5A). We note that this is similar to the dimensionality of locomotor space (participation ratio = 7.94, above), compatible with the idea that RSN activity has a functional complexity that is much smaller than the number of neurons or anatomically defined cell types yet well-matched to behavioral complexity. Second, we revealed the most common relationships between RSN activity and motor kinematics by applying a multi-stage hierarchical clustering procedure to the kinematic modulation vectors (STAR Methods). This identified 8 clusters comprising 9,941 cells (53% of all imaged neurons, percentage of clustered cells was similar across fish: 10th–90th percentile = 42.0%–63%, *n* = 67).

The 8 clusters provide a compact representation of functional diversity within the RSN population. Inspection of kinematic modulation vectors confirmed that cells within a cluster showed similar patterns of activity across swims, whereas different clusters showed quite distinct patterns (Figure 5B). This was also true for an additional ~3,000 “unseen” bouts that were not part of the clustering procedure (swims on right side of yellow line in Figure 5B). A uniform manifold approximation and projection (UMAP) embedding of kinematic modulation vectors showed that the clusters tiled the embedding space with minimal overlap (Figure S5B). However, they were not well separated, suggesting continuous variability in functional properties. Thus, we prefer not to interpret clusters as corresponding to distinct cell types, but instead as *functional archetypes*, which capture the predominant functional properties within the RSN population.

The 8 functional archetypes had distinct anatomical distributions and, in every case, spanned multiple anatomically defined cell types (Figure 5C). Four archetypes exhibited a symmetric distribution between left and right sides of the brain (ar1–3 and 8) (Figure 5C, top row), whereas the remaining 4 (ar4–7) were strongly lateralized, forming 2 mirror-symmetric pairs that labeled left- or right-sided cells (Figure 5C, bottom row). Moreover, we observed substantial functional diversity within anatomical cell types. Thus, cells of the same anatomical type often spanned a broad region of the embedding space and consequently were assigned to different functional archetypes (Figures S6 and S7). For example, both MiT (singly occurring) and RoV3 (multiply occurring) cells associated with ar1–3 plus either ar4 (left sided MiT/RoV3 cells) or ar5 (right-sided cells) (Figure S7). Such variation was observed for both canonical RSNs and the additional cell types labeled by the *u508* transgene, albeit to different degrees. One possibility was that this variability in model-predicted kinematic modulation might be a consequence of regression models being biased by the particular set of swims that occurred concurrent with calcium imaging. However, when we inspected co-imaged subsets of neurons (i.e., cells of the same anatomical type imaged simultaneously in the same focal plane and thus having activity recorded coincident with the same set of swims) we observed similar variability both in functional mapping and raw calcium data, directly supporting functional diversity within anatomical cell types (Figure S7). Taken together, our data supports a continuum of functional properties within and across cell types and suggests that anatomical labels might not be well-suited to understand the operation of RSN populations.

### Functional archetypes provide interpretable relationships between RSN activity and motor kinematics

To understand what features of behavior might be controlled by these functional archetypes, we examined model-predicted activity across bout types as well as model coefficients. We computed mean spiking rates (*μ*) for each archetype and swim bout type (Figure 5D) and focused on the coefficients’ fit in the basis of kinematic modes (Figure 5E, enlarged and more comprehensively labeled in Figure S5D). Although these models had slightly poorer *R*^2^ for most neurons, they aided interpretability by smoothing coefficient weights across correlated predictors.

Activity of the symmetric archetypes ar1–3 varied systematically across locomotor space in a manner that suggested they play distinct but complimentary roles in controlling swim speed (Figures 5D and S5C). For ar1, activity declined monotonically across the kinematic sequence F1 → T2. By contrast, ar2 was most active for swims of intermediate vigor (F3 and F4), with activity declining both for faster (F1 and F2) and more lateralized (T1 and T2) swims. Finally, ar3 was a “fast-swimming” archetype whose activity was strongly biased to F1 and F2 swims. Model coefficients (Figure S5D) indicated that both ar1 and ar3 were positively modulated by TBF, with ar3 encoding a higher range of cycle frequencies than ar1, in accordance with its selective activation during the fastest forward swims. By contrast, ar2 was tuned to frequencies centered around 30 Hz and had large model coefficients related to swim vigor and bout duration but not TBF. All 3 showed minimal sensitivity to swim direction. Neurons assigned to ar1–3 were broadly distributed (Figures 5C and S6), being abundant in the nMLF, which has previously been implicated in forward swimming^22,23,51^ and speed control,^20,21^ as well as in the RoL1 and MiV1 clusters. RoR1, MiR1, and MiR2 were strongly associated with the fast-swimming ar3 archetype. Also associated with ar1–3 were various *u508* cell types, with a small cluster of cells in the Mi2 region (“Mi2 medial”) being a salient feature of ar2.

The asymmetric functional archetypes ar4 and ar5 were active for left- and right-lateralized swims, respectively (Figure 5D and S5C). Model coefficients revealed modulation by kinematics associated with movement direction (notably theta_1, theta_3, and vel_1–4) (Figure S5D). These functional archetypes were active across almost the entire kinematic sequence, including the fastest F1 swims and the most strongly lateralized T2 turns, in accordance with our previous observation that RSN activity is consistently lateralized toward the side of the body from which locomotion is initiated. The notable exceptions were J-turns, which showed minimal ar4/5 activity. Neurons associated with ar4/5 were abundant in ventromedial RoV3 and MiV2 clusters and also included RoM1r and MiT, ipsilateral to swim direction (Figure 5C and S6).

Three functional archetypes, ar6–8, were almost exclusively active during hunting-related J-turns (Figure 5D). All 3 had negative model coefficients for swim vigor, which is suppressed in this bout type, but had positive coefficients for kinematic features that are elevated during J-turns, including half-beat periods and ocular vergence (Figure S5D). A defining characteristic of J-turns is that tail curvature is concentrated toward the tip of the tail^43^ and accordingly, ar6 and ar7, which are active for left and right J-turns respectively, showed positive coefficients for ipsiversive curvature of the caudal-most tail segments (e.g., fcC1). Ar6–8 collectively explained the sparse J-turn recruitment maps (Figure 3), with lateralized *u508* populations “Mi3 lateral” and “Ca1 lateral” being associated with ar6/7, whereas notable cells in the symmetric ar8 archetype included RoM1r/c and RoM2r (Figures 5C and S6).

### Modular encoding of speed, direction, and behavioral context

Next, we sought to validate the model-predicted activity patterns by directly assessing calcium activity and swim kinematics.

**Figure 6:**
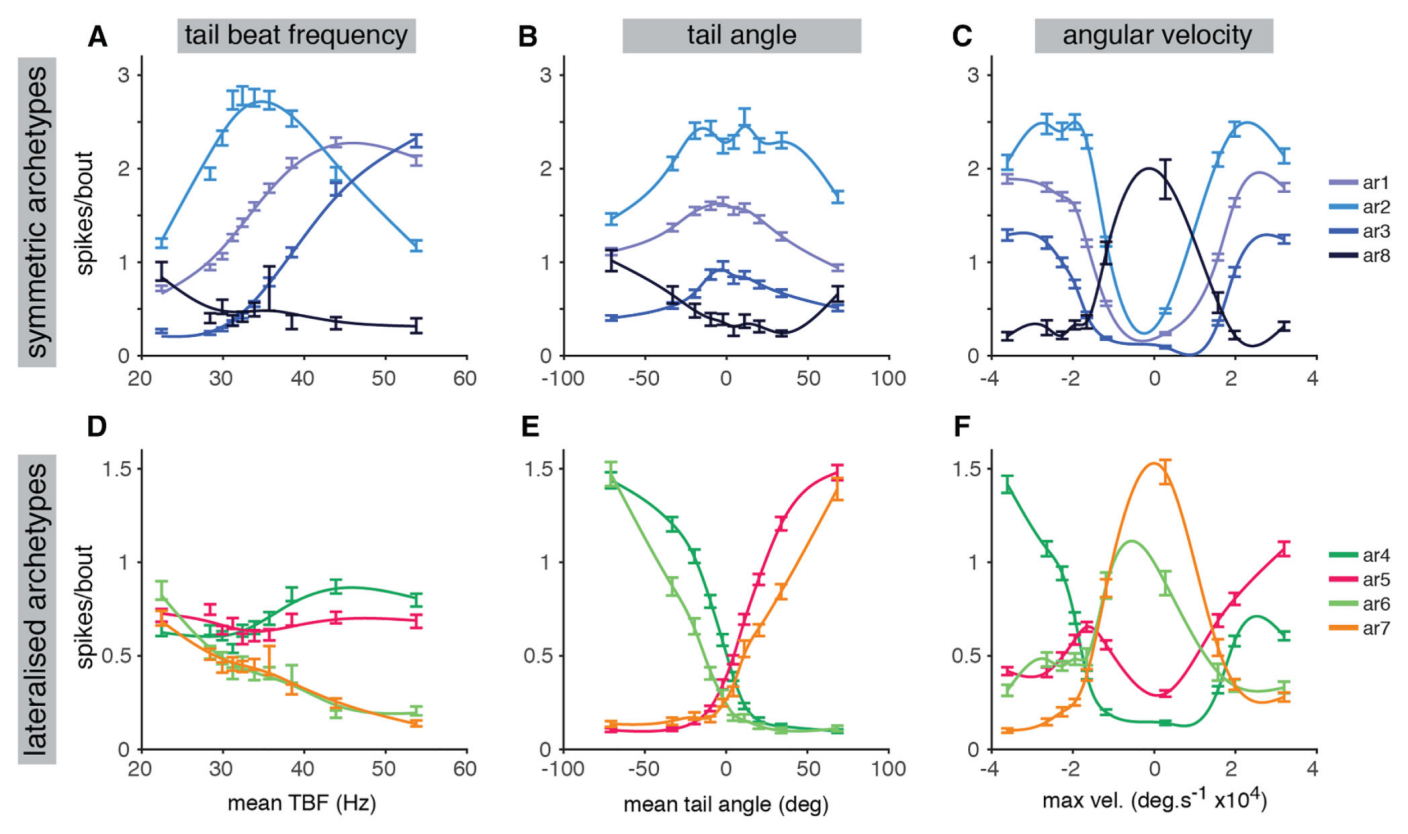
Functional archetypes have distinct kinematic tuning (A–F) Kinematic tuning of neurons belonging to symmetric (A–C) and lateralized (D–F) functional archetypes, showing OASIS-inferred spikes per swim bout as a function of (A and D) mean tail-beat frequency; (B and E) mean tail angle during first 120 ms of the swim; (C and F) peak angular velocity. For each kinematic, 10 bins were defined containing an equal density of data points. Data shows mean ± SEM across neurons assigned to each archetype with superimposed cubic spline fits. Number of cells: *n* = 2,349 (ar1), 1,047 (ar2), 1,405 (ar3), 1,826 (ar4), 1,821 (ar5), 585 (ar6), 677 (ar7), and 231 (ar8).

First, we analyzed neurons associated with the symmetric functional archetypes. As predicted from the GLM models, the activity of neurons assigned to ar1–3 was strongly modulated by cycle frequency, but with each archetype showing a distinct pattern of tuning (Figure 6A). For ar2, activity peaked at 30–35 Hz but declined at both lower and higher frequencies, in agreement with our previous analysis that predicted maximal activity during swims of intermediate vigor (F3 and F4, which have a large PSD peak at ~30 Hz). By contrast, the activity of cells associated with ar1 and ar3 increased monotonically with TBF but the 2 archetypes operated over distinct dynamic ranges. For ar1, activity started increasing from 20 Hz and appeared to plateau around 40 Hz, whereas the fast-swimming ar3 neurons were tuned to a higher range of cycle frequencies, with activity starting above ~30 Hz and increasing thereafter. These patterns aligned with model predictions, which showed a smooth gradient of ar1 activity across the kinematic sequence while ar3 activity was biased toward the fastest swim types. When we assessed tuning with respect to mean tail angle, ar1–3 all displayed peaks close to zero, consistent with the idea that they control locomotor speed but not direction (Figure 6B). The ar8-associated cells showed distinct kinematic tuning. Their activity was greatest at low TBF (Figure 6A), low angular velocity (Figure 6C), and high lateralization (but without a left/right bias) (Figure 6B), consistent with model-predicted recruitment during both left and right J-turns.

Direct analysis of tuning properties also supported the idea that the lateralized functional archetypes (ar4–7) provide independent control of steering. These cells showed approximately rectilinear tuning, wherein activity increased with mean tail angle during ipsiversive swims (i.e., left swims for ar4/6 and right swims for ar5/7) but was minimal during contraversive swims (Figure 6E). Cells in ar4/5 showed little sensitivity to TBF, compatible with the idea that they function across the majority of locomotor space (Figure 6D). By contrast, ar6/7 cells were most active at low TBF and during low angular velocity bouts (Figure 6F), consistent with their predicted role in directional control of J-turns.

### A compact set of functional archetypes support reliable locomotor control

Finally, we modeled descending commands as distributed activation patterns across the 8 functional archetypes. We hypothesized that this low-dimensional command signal would suffice for downstream circuits to decode the intended locomotor output and that, by pooling activity across many cells of each archetype, the readout would be robust to random variation in single-cell spiking.

**Figure 7:**
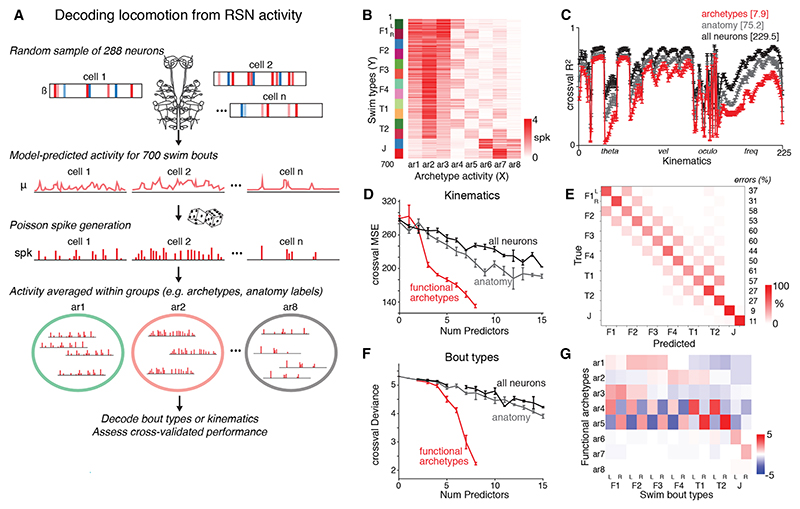
Functional archetypes enable succinct decoding of swim type and kinematics (A) Schematic of decoding process. For each iteration, we pseudorandomly sampled 288 neurons with the same number of each anatomical type as is observed in individual fish. Next, the cells’ best GLM models were used to predict their activity across a random set of 700 swims, evenly sampled across bout types. Using this estimate of *μ*, spikes were emitted according to a Poisson process. Activity was then averaged across neurons belonging to each functional archetype and used to train a linear decoder. This was repeated for 10 iterations and performance evaluated using cross-validation. (B) Example of one iteration. For 700 swims (rows), bout types (color-coded on left) were predicted from functional archetype activity vectors. (C) Decoding performance (quantified as cross-validated R-squared) for motor kinematics. Mean number of predictors selected by LASSO decoder shown in square brackets. (D) Kinematic decoder performance (quantified as cross-validated mean squared error) as a function of number of predictors. (E) Confusion matrix for multinomial LASSO decoder predicting bout type from functional archetype activity. (F) Bout type decoder performance (quantified as cross-validated multinomial deviance) as a function of number of predictors. (G) Coefficients for bout type decoder. C–G show means across 10 decoding iterations; error bars show SEM.

Linear decoders were trained to predict either swim bout type or individual motor kinematics (Figure 7A). For each decoding simulation, we generated a virtual RSN population by sampling 288 neurons from our dataset to match the average number of each anatomically defined cell type observed in the zebrafish brainstem. Using the fitted GLM models for these cells, we predicted their activity for a pseudorandom sample of 700 swim events and simulated spiking variability by having each cell emit spikes according to a Poisson process with the model-predicted rate, *μ* (Figures 7A and 7B). We then decoded swim type or motor kinematics from spiking activity averaged across neurons within each functional archetype and compared this with decoding performance using single neurons or activity averaged within anatomically defined cell types.

We first used multivariate LASSO decoders to predict swim kinematics from RSN activity. Interestingly, different kinematics showed substantial variation in how well they could be decoded (Figure 7C). This might be because some are controlled by latent dynamics within the supraspinal population (not captured by our single-cell encoding models)^54^ or modulated by spinal activity or passive physical processes. Swim laterality, bout asymmetry, and tail velocity were well predicted. Tail angle was accurately decoded for the first and third half-beats (i.e., during activation of axial muscles on the same side of the body as swim direction) but much less so for the second and fourth (when contralateral motoneurons are active). For each type of decoding model, the best cross-validated performance was obtained using most available predictors (mean degrees of freedom [cf. total available predictors], for single-neuron decoder: 229.5 [from 288]; anatomical types: 75.2 [from 78]; functional archetypes: 7.9 [from 8]), and decoders trained using many single neurons performed better than those using fewer anatomical types or functional archetypes. This indicates that some behaviorally relevant RSN activity is not captured by the 8 archetypes, but they nonetheless provided good decoding for a broad range of kinematics. Importantly, kinematics were much more succinctly represented in the activity of functional archetypes than anatomical cell types or individual neurons. We showed this by evaluating decoding performance as a function of the number of predictors in the model and observed that cross-validated error decreased most rapidly when decoding from archetypes (Figure 7D).

The activity of functional archetypes was also effective in predicting bout types, which we showed by training multinomial LASSO decoders to predict the 14 bout labels (Figure 7E). Moreover, classification errors almost always predicted swims that were similar to the correct type (i.e., adjacent in the kinematic sequence and of the same laterality), and the kinematically distinct J-turns had very low error rates. Again, cross-validated error decreased most rapidly when decoding from functional archetypes (Figure 7F). These decoding analyses therefore support our hypothesis that functional archetypes provide a succinct and robust code for controlling locomotion.

Overall, this study suggests that varying activation patterns across a small number of functional archetypes provide low-dimensional descending commands that control locomotion. The decoding model coefficients summarize a simple functional logic (Figure 7G). Thus, across the majority of locomotor space, ar1–3 control swim speed (thereby specifying the position of a swim within the kinematic sequence), while parallel activation of either ar4 or ar5 imparts directionality. By contrast, J-turns are controlled by an independent modular code in which ar8 is consistently active, and steering is mediated by additional activity of ar6 or ar7. In this model, ar8 provides a symmetric scaffold on which these swims are built but, due to co-activation with ar6/7, ar8 is dispensable for statistical decoding (Figure 7G).

## Discussion

In this study, we present evidence that reticulospinal population activity is organized into a small number of functional archetypes whose combinatorial activity provides robust descending commands to instruct locomotion. Despite broad and smoothly varying patterns of RSN activity across much of locomotor space, single-neuron modeling revealed a low-dimensional organization that could be summarized by 8 functional archetypes. The vast majority of swims were encoded by 5 archetypes, with multiplexed encoding of swim speed and independent encoding of swim direction, while 3 additional archetypes appeared to be specialized for hunting-related swims. Overall, our data support a modular organization for supraspinal control and reveal additional stratification according to behavioral context.

### Supraspinal activity is low-dimensional and behavioral context specific

Our description of functional archetypes accords with a broad body of evidence that motor control has a low-dimensional organization. For instance, there are long-standing ideas that the nervous system can construct diverse movement patterns using linear combinations of a small set of muscle synergies,^55–58^ that complex three-dimensional reaching movements in primates associate with low-dimensional cortical activity^54,59^ (but see also Marshall et al.^60^), and that in *Drosophila* diverse movements are controlled by modules of descending neurons that individually control specific motor primitives that combine to compose a complete behavior.^61–64^ Explanations for low dimensionality include the idea that it reduces the daunting complexity of motor control from a large number of degrees of freedom (i.e., hundreds of motor units) to a far smaller set of “basis functions” that provide near-optimal patterns of muscle activation.^65^ Relatedly, a modular organization might reduce interference between simultaneously incompatible actions.^66,67^ In the context of descending control, it is notable that RSNs have large, fast-conducting axons that can fire at hundreds of hertz,^4^ including in larval zebrafish,^40,68^ and thus low dimensionality may relate to the need to limit energy expenditure by compressing motor commands through a relatively small descending population. Finally, as supported by our decoding analysis, a small number of control channels, instantiated across multiple neurons, may improve the robustness of information transmission in the face of variation in single-cell properties and a stochastic spike-generation process.

While the dimensionality of RSN activity appeared well-matched to behavior (in both cases the participation ratio was around 8), these estimates are likely to represent lower bounds. Although calcium imaging allowed us to monitor a large population of cells, it has limited sensitivity and temporal resolution. Thus, we could not resolve firing rate modulation during individual bouts, such as the start, maintain, and stop activity that temporally delimits swim episodes in lamprey,^7^ nor tonic versus phasic firing that is thought to be relevant for spinal decoding of descending commands.^68,69^ Additional complexity also stands to be uncovered by expanding our survey of behaviors. Zebra-fish generate 13 basic bout types,^13^ and we examined only a subset of these, notably excluding escape swims^18,19,44^ and capture swims, which have distinct kinematics and presumably descending control.^42,46^ Nonetheless, our data indicate that the combinatorial action of small set of modules is a fundamental aspect of supraspinal control and that mutually exclusive sets of modules operate in different behavioral contexts. This reveals an organizational logic that is a hybrid between a switching code, with dedicated labeled lines for specific movement types, versus a set of universal modules that control movement kinematics across the entirety of locomotor space.

### Multiplexed descending control of swim speed

Three functional archetypes (ar1–3) appear to have complimentary roles in controlling swim speed. Previous studies have implicated the nMLF in speed control in both larval^20^ and juvenile/adult^21^ zebrafish, and accordingly ar1–3 include the majority of cells in this midbrain nucleus. However, speed encoding was not limited to this region but instead appeared broadly distributed across the tegmentum. Additional populations with a high prevalence in ar1–3 include MiV1, RoL1, and RoR1, which were previously associated with forward swimming,^17,23,24^ and in the case of RoL1 are thought to mediate avoidance responses to homeostatic threats and acute nociceptive stimuli.^27,28^ The MiR1 and MiR2 cells, which are active during loom-evoked escapes,^26^ are predominantly assigned to ar1 and ar3, suggesting they contribute to the high TBF characteristic of this swim type.

Behaviorally, zebrafish larvae modulate their speed by varying cycle frequency, amplitude, and bout duration.^20,70^ The kinematic tuning of ar1 and ar3 suggests these archetypes modulate TBF, with population activity increasing monotonically with frequency before saturating. In support, electrophysiology has shown similar patterns of firing rate modulation for single RSNs in lamprey^71^ and zebrafish.^17^ However, the 2 archetypes operated over different dynamic ranges. For ar1, most modulation occurs from ~20–40 Hz, compatible with a role in controlling routine exploratory swims and those evoked by whole-field motion,^20^ whereas ar3 is tuned to higher speeds, ~ 30–50 Hz, characteristic of burst swims and the propulsive phase of C-start escapes.^13,26,41^ In lamprey, neurons in middle rhombencephalic reticular nuclei (MRRN) and the posterior rhombencephalic reticular nuclei (PRRN) modulate their firing rates across slow versus fast cycle frequencies respectively,^71^ and glutamatergic neurons in the lateral paragigantocellular nucleus of mammals appear to specifically command high-speed locomotion.^9^ This suggests multi-channel control of cycle frequency might be a conserved feature of reticulospinal organization, perhaps enabling finer-scale speed control than could be realized by a single population with limited dynamic range.

In the slowest swimming regime (^10 mm/s), increases in average speed are controlled by extending bout duration (i.e., more oscillatory cycles at ~30 Hz), with a concomitant reduction in interbout duration.^20^ Here, it is notable that ar2 showed “bandpass” tuning that peaked around 30 Hz (a frequency with high spectral power across all swim types) and had a high encoding model coefficient for bout duration. Thus, our data support a multiplexed control architecture in which ar2 may modulate bout duration and ar1 and 3 modulate cycle frequency, together enabling larvae to adjust their swim speed over a broad dynamic range.

How might these functional archetypes interface with spinal circuits? Changes in swim speed (specifically TBF) are accomplished by topographically ordered recruitment of motoneurons, accompanied by switches between active sets of spinal interneurons.^72–74^ At adult stages, 3 modules of recurrently connected V2a interneurons and motoneurons are progressively recruited at slow, intermediate, and fast speeds,^75,76^ and it has been hypothesized that descending commands may be segregated into parallel streams that selectively target each module.^77^ As the molecular signatures of these spinal modules can already be recognized at larval stages,^78^ we hypothesize that ar1–3 represent these parallel descending signals. It will be a priority of future studies to elucidate synaptic connectivity between functionally defined descending axons and specific spinal targets. Because changes in speed are associated with different distributions of bending along the trunk and tail,^20,41,79^ and control of bout duration appears to be biased toward rostral segments,^80^ synaptic connectivity from ar1–3 axons is likely also patterned along the anterior-posterior axis. However, specific synaptic connectivity may not be the only mechanism to decode parallel, speed-tuned descending signals. At least for motoneurons, intrinsic properties have been shown to influence speed-dependent recruitment by affecting the temporal integration of synaptic inputs.^68^ Here, it is notable that neurons in lamprey PRRN that are recruited at fast speeds display phasic firing (as opposed to tonic firing of MRRN neurons).^71^ If this is also the case for ar3, this could provide a temporal code for recruitment of dorsal spinal motoneurons with fast membrane time constants.^68^

### RSN control of steering

Two functional archetypes, ar4 and ar5, appeared to act independently of speed commands to control swim direction across most of locomotor space. In agreement with previous studies, cells assigned to these archetypes were concentrated in ventro-medial RSN clusters and showed rectilinear activity that increased with steering toward the ipsilateral side.^23–25^ In contrast to early studies suggesting widespread modulation of RSNs during turns,^81,82^ ar4/5 are mostly restricted to the RoV3 and MiV2 clusters (and to a lesser extent MiV1). These neurons express *vsx2*^17^ and might be homologous to Vsx2-positive Gi neurons in the rostral medulla of mammals that mediate turning when unilaterally activated.^10,11,83^ While asymmetric activity was greatest during large amplitude turns, at least some asymmetry was apparent across locomotor space, including for minimally lateralized forward swims. This suggests that ar4/5 specify the side of spinal cord from which locomotion is initiated. Supporting this, ablation of RoV3, MiV1, and MiV2 cells has been shown to abolish the directional bias of the first tail undulation of forward swims evoked by phototactic or optomotor stimuli.^24^ Previous studies have argued that RoV3, MiV1, and MiV2 play a universal role in controlling turns, based on analysis of spontaneous behavior and turns evoked by phototactic, optomotor, and dark flash stimuli^23,24^ as well as loom-evoked escapes.^26^ It was therefore surprising that these neurons showed minimal recruitment during hunting-related J-turns, which we found are instead associated with an independent set of reticulospinal modules.

In accordance with independent supraspinal control of propulsion and steering, recent studies have suggested similar modularity in the spinal cord.^84^ Specifically, changes in direction are associated with ipsiversive increases in motoneuron burst duration that are independent from timing features (frequency, left-right phasing, and rostrocaudal phase lag). Direction-selective V2a-B interneurons are proposed to mediate these effects^84^ and are a strong candidate for receiving descending commands from ar4/5 axons. Turn-associated V2a RSNs have also been shown to make direct synaptic connections onto spinal motoneurons.^25^

### Independent supraspinal control of locomotion during hunting

A key finding of our study is that the modular organization of reticulospinal activity is stratified by behavioral context, with 3 functional archetypes specialized for hunting. By using a virtual prey-capture assay and tracking naturalistic, tail-free behavior,^29,30^ we could relate RSN activity to execution of the specialized swims that larvae deploy during prey tracking, and which would be very challenging to recognize using fictive recordings. Early descriptions of J-turns described their distinctive pattern of unilateral bending confined to the most caudal regions of the tail and hypothesized this might result from bilateral activation of rostral trunk musculature (rostral stiffening) combined with lateralized activation of spinal circuits on the turning side.^43,85^ Our findings may be compatible with this model. Specifically, ar8 was active for all J-turns and included bilateral activity in the newly described RoM2r cells. Preliminary experiments indicate that these neurons project ipsilaterally along almost the entire length of spinal cord (data not shown). We speculate that ar8 provides bilateral descending drive to axial muscles that stiffens the tail and provides a scaffold for prey-tracking swims. J-turns show graded lateralization such that body reorientation scales with prey azimuth.^45^ Our data suggest steering is controlled by ar6 and ar7, which, like ar4/5, showed rectilinear activity that progressively increased with ipsiversive turn angle. Ar6 and ar7 are primarily composed of laterally located cells that are labeled by the *u508* transgene but not readily backfilled from the spinal cord. While some of these cells, located in rhombomeres 5–6, almost certainly include abducens internuclear neurons involved in the saccadic eye movements that accompany J-turns,^86^ others are likely descending neurons that project in the lateral longitudinal fasciculus but whose bulbar and/or spinal targets are not yet known.

Why should an independent set of supraspinal modules be engaged during hunting? One explanation may relate to the need for greater precision to accurately target moving prey. A hint that supports this idea is that activity of ar6/7 increased more linearly with turn angle as compared with ar4/5. During hunting, changes in head yaw show a precise linear relationship with prey azimuth,^45,48,87^ and this might be achieved by this dedicated directional control system. We recently discovered that the specialized saccadic eye movements that zebrafish use to foveate prey are controlled by a dedicated premotor pathway that recruits a specialized subset of oculomotor neurons.^86^ A priority for future work will be to map the afferent and efferent connectivity of ar6–8 to determine whether they similarly engage specialized spinal targets, how brainstem circuits interact to coordinate eye and body movements during hunting,^48^ and how the forebrain circuits that command hunting state^34^ interface with these supraspinal modules.

### Functional heterogeneity at cellular resolution

The modular organization of supraspinal control that we describe was based on analysis of single-cell function, and most functional archetypes did not align neatly with anatomical labels. The distribution of motor commands across broad, delocalized cell populations aligns with findings in lamprey^7^ and mouse^2^ and implies considerable redundancy that seems compatible with the fact that laser-ablations in zebrafish have typically failed to significantly impair swimming behavior.^17,88,89^

As well as a one-to-many mapping between functional archetype and anatomical cell type, we also found that specific anatomical types (including singly occurring neurons) showed substantial variation in kinematic encoding. Previous studies have often averaged activity across cells according to anatomical label,^17,26^ but variability between such cells (and across trials) is evident in several published datasets.^17,23–25,51^ In recent years, stratification of neurons by gene expression, neurotransmitter phenotype, and/or connectivity has been successful in ascribing specific functional roles to subsets of RSNs,^3,6^ and approaches such as single-cell transcriptomics, *in situ* hybridization, and projection-specific transgenic lines^90^ should be combined with analysis of single-cell function in future studies. However, it may be challenging to explain functional heterogeneity using such methods, especially for the singly occurring RSNs that presumably arise from a consistent, genetically specified, developmental program. Indeed, mirroring our findings, recent studies in zebrafish have reported substantial functional diversity within groups of hypothalamic neurons defined by specific peptidergic phenotypes^28^ and within transcriptionally defined clusters in the optic tectum.^91^ Whatever the source of such variability, these studies and ours imply that robust functional properties can be encoded across broad networks of neurons, despite substantial molecular-genetic diversity.

### Flexible supraspinal control across development and internal state

Here, we took advantage of the *u508* transgene to survey activity across the canonical set of larval RSNs,^14,35,36^ which also constitute the core of the adult descending control system.^21,37^ However, additional spinally projecting neurons include a subset of cells expressing *vsx2* (V2a neurons), which are capable of evoking locomotion and extend broadly throughout the pontomedullary hindbrain.^15,92^ An elegant study^17^ revealed a “chronotopic” organization within the *vsx2* population, wherein early born cells are associated with “crude” movements while those born later in development give rise to parallel descending pathways that control “finer” swim types. Hunting involves precise motor control and emerges at relatively late stages (from ~4 dpf) and we found was associated with rather sparse activity within the canonical reticulospinal population. Therefore, it seems probable that later-born *vsx2*-positive cells are additional constituents of the supraspinal modules that command these specialized swims.

Finally, we note that our modeling approach assumed that neurons have time-invariant encoding properties. However, the reticulospinal tegmentum receives a broad array of neuromodulatory and neuropeptidergic afferent inputs,^4,5^ including in larval zebrafish,^27,28^ which are believed to underlie acute changes in RSN excitability^40^ as well as longer-lasting reconfiguration in accordance with internal states.^93,94^ Conceivably, such modulation might switch singly occurring cells between functional archetypes. Moreover, spinal decoding of descending commands is also flexible, being subject to neuromodulation,^75,95–97^ local synaptic plasticity mechanisms, and sensory feedback.^98–100^ Future work will build on the framework we have developed to extend cellular-resolution models of supraspinal control to include dynamic changes in population encoding and spinal decoding, including during transitions between behavioral states.

## Resource Availability

### Lead contact

Further information and requests for resources and reagents should be directed to and will be fulfilled by the lead contact, Isaac H. Bianco (i.bianco@ucl.ac.uk).

### Materials availability

The transgenic line used in this study is available from the lead contact on request.

### Star★Methods

#### Key Resources Table

**Table:** 

REAGENT or RESOURCE	SOURCE	IDENTIFIER
**Chemicals, peptides, and recombinant proteins**		
Dextran, Texas Red, 3000 MW, Lysine Fixable	Invitrogen	D3328
**Deposited data**		
Processed calcium imaging and behaviour data		Mendeley Data: https://doi.org/10.17632/v8wd82hkg9.2
**Experimental models: Organisms/strains**		
Tg(pvalb6:KalTA4)u508Tg	Antinucci et al.^34^	ZFIN: ZDB-ALT-200519-9
Tg(UAS:GCaMP6f;cryaa:mCherry)icm06	Bohm et al.^52^	ZFIN: ZDB-ALT-160119-5
**Software and algorithms**		
MATLAB 2021a	Mathworks	https://uk.mathworks.com/products/new_products/release2021a.html
UMAP (MATLAB implementation)	Meehan et al.^105^	https://uk.mathworks.com/matlabcentral/fileexchange/71902-uniform-manifold-approximation-and-projection-umap
Psychophysics Toolbox	Brainard^101^	http://psychtoolbox.org/
glmnet package for MATLAB	Qian et al^103^	https://hastie.su.domains/glmnet_matlab/
LabView	National Instruments	https://www.ni.com/en/shop/labview.html
Original MATLAB scripts	This paper	Mendeley Data: https://doi.org/10.17632/v8wd82hkg9.1

### Experimental Model and Study Participant Details

#### Zebrafish lines and care

For all experiments, we used zebrafish larvae carrying two transgenes Tg(pvalb6:KalTA4)u508^34^ and Tg(UAS:GCaMP6f;cryaa: mCherry)icm06^52^ as well as the mitfa^53^ skin-pigmentation mutation in homozygosity and lines were maintained in the Tübingen background. For brevity, the genotype is referred to as *u508*:GCaMP6f. Animals were reared on a 14/10 h light/dark cycle at 28:5° C. All larvae were fed *Paramecia* from 4 dpf onward. The sex of the larvae is not defined at the early stages of development used for these studies. Experimental procedures were approved by the UK Home Office under the Animals (Scientific Procedures) Act 1986.

### Method Details

#### 2-photon calcium imaging and behavioral tracking

*u508*:GCaMP6f larvae were tethered in 2% low-melting point agarose gel in a 35 mm petri dish lid and sections of gel were carefully removed using an opthalmic scalpel to allow free movement of the eyes and tail below the swim bladder. Larvae were allowed to recover overnight before functional imaging at 6 or 7 dpf. Imaging was performed using a custom-built multiphoton microscope [Olympus XLUMPLFLN × 20 1.0 NA objective, 580 nm PMT dichroic, bandpass filters: 510/84 (green), 641/75 (red) (Semrock), R10699 PMT (Hamamatsu), Chameleon II ultrafast laser (Coherent)] at 920 nm with laser power at sample of ~ 10 mW. Images (0.67 *μ*m/px) were acquired by frame scanning at 4.8 Hz and we imaged an average of 16 focal planes with an axial (‘z’) spacing of 5 *μ*m.

We used two projectors to present visual stimuli. The first (Optoma ML750ST) back-projected stimuli onto a curved screen placed in front of the animal (viewing distance 35 mm), while the second (AAXA P2 Jr) projected images onto a diffusive screen directly beneath the chamber (viewing distance 5 mm). Wratten filters (Kodak, no. 29) were placed in front of both projectors to prevent visual stimuli interfering with fluorescence detection. Visual stimuli were designed in MATLAB using Psychophysics Toolbox^101^ and presented on a uniform grey background. Prey-like moving spots comprised 6° or 12° bright or dark spots (Weber contrast +1 or -1 respectively) moving at 30°/s either left → right or right → left across 152° of frontal visual space. Stimulus design took account of distortion caused by the curved screen to ensure constant angular velocity and angular size from the point of view of the fish. For dark flashes, both projectors were switched to zero pixel value for 3 s. Looming stimuli comprised expanding dark spots (Weber contrast -1) that simulated an object approaching at constant velocity (10°–70°, L/V 490 ms).^102^ Optomotor stimuli comprised drifting sinusoidal gratings (wavelength 10 mm, velocity 10 mm/s, Michelson contrast 1) presented from below and moving in four cardinal directions with respect to the animal. We also generated a brief (20 ms) mechanosensory water puff stimulus using a micropipette placed immediately adjacent to the left ear of the animal. However, this did not reliably evoke escape-like swim bouts, perhaps indicating the stimulus intensity was too weak. For all experiments, stimuli were presented in a pseudo-random sequence with 30 s interstimulus interval during which a uniform grey screen was shown.

Eye movements were tracked at 60 Hz under 720 nm illumination using a FL3-U3-13Y3M-C camera (Point Grey) that imaged through the microscope objective. Tail movements were imaged at 420 Hz under 850 nm illumination using a sub-stage GS3-U3-41C6NIR-C camera (Point Grey). Horizontal angular eye position and tail posture (extracted as 13 x-y coordinates equally spaced along the length of the tail) were computed online using machine vision algorithms.^30^ Microscope control, stimulus presentation, and behaviour tracking, were implemented using LabVIEW (National Instruments) and MATLAB (MathWorks).

#### Dextran labelling

Reticulospinal neurons (RSNs) were retrogradely labelled by spinal cord injection of dextran-conjugated dye as previously described.^18^ Briefly, larvae were anaesthetised with 0.02% tricaine (MS-222, Sigma) and Texas Red-dextran (3000 MW, Invitrogen) dissolved in distilled water (40 mg/ml) was pressure-injected into the rostral spinal cord at somite 12 using a fine glass micropipette. Larvae were allowed to recover overnight before multiphoton imaging (800 nm, 6.5 mW at sample).

#### Analysis of behavioural data

##### Motor kinematics

Raw behavioural tracking data comprised the horizontal angular position of the two eyes and 13 x-y centroids defining the midline of the tail (example tail skeletons in [Figure 1B]). Consecutive centroids define tail segments and vectors of 11 inter-segment angles were computed for each time-point. Angle–time matrices were interpolated onto a uniform timebase at 1000 Hz and smoothed in 2D using a 2-segment-by-7-ms filter. Next, we computed the cumulative sum of inter-segment angles, *γ*, which was filtered (MATLAB sgolayfilt, order=3, framelength=9) and median subtracted. Thus, changes in tail posture are represented as the time-varying cumulative bend angle along the anterior-posterior axis of the tail (depicted in [Figure 2A]): *γ*_*s;t*_, for cumulative inter-segment angle *s* at time-point *t*. To identify swim bouts, we first estimated tail angular velocity, *v_t_* by differentiating *γ*_11;*t*_, taking its absolute value and filtering (40 ms box-car). We also computed the envelope, *f_t_*, as the maximum absolute value of *γ*_11;*t*_ within a 9 ms sliding window. The start of swim bouts were identified at time-points where *v_t_* > 800 deg/s and *f_t_* > 7 deg, and the end of swim bouts were defined when *v_t_* < 200 deg/s and *f_t_* < 10 deg. Bouts less than 61 ms in duration were excluded.

For each swim bout we extracted 152 kinematic features, listed in Table S1. Swim vigour was estimated from *v_t_* as either its maximum value during the bout (vigmax) or integral over the first 120 ms of the bout (vig120). An estimate of turning was provided by intcum60ms, the integral of *γ*_11;*t*_ over the first 60 ms of the bout.^24^ To quantify asymmetry in tail oscillations, we computed two measures: morphAI2 is the mean value of *γ*_11;*t*_ over the first 120 ms of the bout. morphAI is computed as (*nL* − *nM*)=120, where *nL* is the duration (in ms) for which *γ*_11;*t*_ has the same sign as intcum60ms, and *nM* is the duration for which it has the opposite sign. This metric thus ranges from +1 (a swim bout where the tail bends exclusively towards one side for the first 120 ms) to −1. Zero would indicate a symmetric (i.e. forward) swim. max_angl describes peak cumulative bend angle during the bout and is the (signed) maximum of *γ*_11;*t*_; max_vel describes peak angular velocity and is the (signed) maximum of its derivative.

We identified individual half-beats (leftwards and rightwards excursions of the tail) by finding the maxima and minima of *γ*_9;*t*_. This ninth cumulative inter-segment angle was less prone to tracking noise than the eleventh. Half-beat duration (period) was computed as the interval between velocity maxima/minima surrounding each of these angular position peaks. For the first four half-beats of each swim we then extracted the amplitudes (theta) and angular velocities (vel) for cumulative inter-segment angles 5–11. The sign of the first half-beat, specifically theta_1_s9, was used to define bout laterality (left or right). Because J-turns involve bending localised to the caudal tip of the tail, for half-beat 1 we computed the fraction of total tail bend angle, theta_1_s11, that could be attributed to rostral (s1–5), middle (s6–8) or caudal (s9–11) inter-segment angles. ratio_tp1 is the ratio of the amplitude of the second half-beat (i.e. first ‘trough’) to the first half-beat, at s11. ratio_period2v1 is the ratio of period2 to period1. ratio_theta2v1 is the ratio of the amplitude of the third half-beat (i.e. second tail excursion in direction of the bout) to the first half-beat, at s11.

Instantaneous tail-beat frequencies were computed as the reciprocals of cycle periods, i.e. the intervals between successive maxima (or minima) of *γ*_9;*t*_ for right (or left) lateralised swims. We then computed the mean (mean_TBF) or maximum (max_TBF) of the set of instantaneous tail-beat frequency estimates. We also performed frequency decomposition by applying the fast Fourier transform (FFT) to *γ*_9;*t*_. Power at frequencies from 1–70 Hz was quantified (fourierpsd).

Zebrafish perform saccadic eye movements coincident with swim bouts, including convergent saccades that are coordinated with J-turns.^48^ We therefore computed median eye position before the start (− 120→− 70 ms) and after the end (+ 50→ + 100 ms) of each swim bout and analysed the change in eye position (delta) and post-bout eye position (post). Vergence (V) was calculated as the difference between left (L) and right (R) eye position.

Finally, to clip outlying values that might be due to tracking errors, we applied 99% winsorization to all motor kinematics, based on the distribution of raw values from the entire dataset across *N* = 67 larvae.

##### Analysis of swim kinematics and identification of swim bout types

For most analyses, kinematic features were standardised by z-scoring. This was performed separately for swims from each animal to account for differences in tracking and behaviour across larvae. We then combined standardised data across animals to compute the principal components of the kinematics (MATLAB pca with singular value decomposition (SVD) algorithm).

Dimensionality was estimated from the eigenspectrum of the kinematic covariance matrix by computing the participation ratio:

(Equation 1) $$d=\frac{{\left(\underset{i}{\sum }\lambda_{i}\right)}^{2}}{\underset{i}{\sum }\lambda_{i}^{2}}$$

where *λ_i_* is the *i* th eigenvalue. If all variance were concentrated in one dimension (i.e. only *λ*_1_ > 0), then *d* = 1, whereas if variance were evenly distributed across all *M* eigenvectors (i.e. all eigenvalues are equal), then *d* = *M*. Thus, the participation ratio is an intuitive and continuous measure of dimensionality that we found in practice approximates the number of principal components required to explain 75 − 80% variance.

To identify groups of swims with similar kinematic feature vectors, we implemented a multi-stage clustering procedure as follows:

1. *Batch-clusters*. We applied hierarchical agglomerative clustering^30^ to bout data represented in the space of the first 20 principal components of the kinematics (correlation distance metric, threshold = 0:9). For computational tractability, data from each larvae was clustered separately to produce a library of 2424 ‘batch-clusters’.
2. *Merging of batch-clusters*. Next, these fish-specific batch-clusters were merged. To do this, we computed the distance between all pairs of batch-clusters, quantified as the Pearson’s correlation of their centroids scaled by the median intra-cluster correlation distance of the more compact cluster. The resulting distance matrix was subjected to hierarchical clustering with the dendrogram cut at its longest link. To obtain a robust solution, this process was repeated multiple times, with kinematic data represented in the space of the first *m* principal components, where *m* ∈ [5; 15]. This enabled us to construct an evidence accumulation matrix, *EA*, quantifying the fraction of iterations for which each batch-cluster pair was assigned to the same cluster. Finally, 1 − *EA* defined a distance matrix on which we performed hierarchical clustering, cutting the dendrogram at the longest link. In this way, all 2424 batch-clusters were combined into 20 preliminary clusters.
3. *Cluster curation*. Clusters were ‘trimmed’ by removing swim bouts located beyond the 70th percentile of the distribution of correlation distances to the cluster centroid. By manual inspection we then eliminated small and heterogeneous clusters. Clusters containing a mixture of swim lateralities (defined by the sign of theta_1_s9), were split such that left- and right-directed swims were allocated to separate clusters. In this way we obtained 14 clusters.
4. *Swim label assignment*. Finally, we evaluated the correlation distance between the kinematic vectors of every swim and the centroids of the clusters. Swim events were assigned to the closest cluster of the same laterality below a threshold distance (set to the same threshold as used in the previous curation stage). This resulted in 73,937 swim events being assigned one of 14 bout type labels from an initial dataset of 141,831 swims (from 67 fish). Swims that were not assigned a label were marked as unclassified (UC).

##### t-distributed Stochastic Neighbour Embedding (t-SNE)

To visualise swim bout space, t-SNE was used to generate a 2D embedding of swim bouts. We subjected bout data, in the space of the first 20 principal components of motor kinematics, to t-SNE (MATLAB tsne, Algorithm=barneshut, Distance=correlation, NumDimensions=2, Perplexity=30, Standardize=1, Theta=0.5). The 2D embedding was then colour-coded using the bout type labels assigned by the clustering process described above, or according to the values of specific kinematics.

##### Calcium imaging data analysis and activity inference

Motion correction of fluorescence imaging data was performed as per.^30^ Neurons were manually segmented as binary image masks from mean time series projections of each focal plane. The fluorescence time series of each cell was initially computed as the mean value of pixels belonging to the corresponding binary mask for each imaging frame, assigned to a time-point corresponding the midpoint of that frame scan. For frames where motion error exceeded 4.7 *μ*m, pixel values were replaced by interpolation. This initial time series estimate was then detrended, to correct for slow variations in fluorescence, and standardised by (1) subtracting baseline fluorescence, estimated as the 50th percentile of pixel values within a 150 s sliding window, and (2) dividing by the standard deviation of the calcium signal baseline, estimated by Gaussian fit using the OASIS^38^ subfunction estimate_baseline_noise. Next, we inferred a spiking process underlying the calcium fluorescence using OASIS to fit a first-order autoregressive model (deconvolveCa, options= (ar1, thresholded, optimise_b)). Spikes were summed across the duration of each swim bout and spike count estimates were rounded to the nearest integer for Poisson regression.

##### RSN recruitment

Activity of RSNs across the space of bout types (Figure 3) was assessed by first computing each cell’s mean spike count for each of the 14 bout type labels and then computing the mean of these vectors across cells belonging to each anatomical label. Mann Whitney U-tests (MATLAB ranksum) were used to assess lateralisation by comparing the distributions of cells’ mean spike counts for right-versus left-sided neurons of each anatomical type.

The similarity of recruitment patterns was assessed by computing the Pearson’s correlation (MATLAB corrcoef) of bout type vectors and cell type vectors ([Figure 3A]). We applied principal component analysis to cell type vectors (excluding data for J-turns) and generated maps of the loadings (i.e. coeffs across cell types, [Figure 3C]) and plots of the scores of PC1–3 across bout types ([Figure 3D, Figure S4D]).

#### Generalised linear regression modelling

##### Poisson Regression

We used a generalised linear regression framework to model individual RSNs. We assumed that each neuron’s spiking activity was statistically independent across swim bouts, with the estimated neuronal spike counts during the *i_th_* bout, *y_i_*, following a Poisson distribution with mean, *μ_i_*:

(Equation 2) $$P\left(y_{i}|\mu_{i}\right)=\frac{\mu_{i}^{y_{i}}e^{-\mu_{i}}}{y_{i}!}$$

This bout-dependent mean was determined by the swim bout’s kinematics according to

(Equation 3) $$\mu_{i}=E\left(y_{i}|x_{i}\right)=e^{x_{i}\beta +\beta_{0}}$$

where *x_i_* is a row vector of kinematic predictors (with cardinality *p*), *β* is a column vector of model weights (with cardinality *p*), *β*_0_ is the model’s bias, and the exponential function relating mean spike counts and motor kinematics is the canonical inverse link function for Poisson regression. For notational brevity, we introduce *Y* to denote the *n* -dimensional column vector of spike counts across bouts and *X* to be the *n* × *p* matrix of swim kinematics across bouts, where *n* is the number of bouts. The log-likelihood function of the Poisson regression model is correspondingly given by:

(Equation 4) $$e\left(\beta ,\beta_{0}\right)=\log P\left(Y|X,\beta ,\beta_{0}\right)=\sum_{i=1}^{n}\left(y_{i}\left(x_{i}\beta +\beta_{0}\right)-e^{x_{i}\beta +\beta_{0}}-\log\left(y_{i}!\right)\right)$$

Similarly, the Poisson deviance is

(Equation 5) $$D\left(\beta ,\beta_{0}\right)=2\left(\ell_{sat}-\ell \left(\beta ,\beta_{0}\right)\right)$$

where *ℓ_sat_* is the log-likelihood of the saturated model where *μ_i_* = *y_i_*.

##### Naïve Elastic Net Regularization

We used a regularised regression procedure to fit models relating neuronal spiking to swim kinematics. Our first class of models used ‘naïve’ elastic net regularised regression,^49^ which minimizes the negative log-likelihood plus a penalty term that interpolates between the *ℓ*^1^-norm and *ℓ*^2^-norm of the coefficients:

(Equation 6) $$\left(\hat{\beta },{\hat{\beta }}_{0}\right)={\text{argmin}}_{\beta ,\beta_{0}}\left(-\frac{1}{n}\ell \left(\beta ,\beta_{0}\right)+\lambda \left(\alpha \|\beta {\|}_{1}+(1-\alpha )\|\beta {\|}_{2}^{2}/2\right)\right)$$

where the *ℓ*^1^-norm and (squared) *ℓ*^2^-norm are given by $\|\beta {\|}_{1}=\Sigma_{j=1}^{p}\left|\beta_{j}\right|\;\text{and}\;\|\beta {\|}_{2}^{2}=\Sigma_{j=1}^{p}\beta_{j}^{2}$, *λ* sets the regularization strength, *α* ∈ [0; 1] sets the fraction of the penalty assigned to the *ℓ*^1^-norm, and $\hat{\beta }$ and ${\hat{\beta }}_{0}$ are the estimated regression weights and bias. We used the glmnet package^103^ and *k* -fold cross-validation (typically with *k* = 10) to simultaneously optimise *α* and *λ*.

We also fit ridge regression models by applying the procedure described with an *α* = 0 constraint.

##### Relaxed Elastic Net Regularization

The naïve elastic net is known to cause excessive coefficient shrinkage and model bias^49^ and we found that better model performance was obtained through ‘relaxed’ elastic net regularisation (following a procedure inspired by^50,104^). We again began by running elastic net regularised regression:

(Equation 7) $$\tilde{\beta }={\text{argmin}}_{\beta ,\beta_{0}}\left(-\frac{1}{n}\ell \left(\beta ,\beta_{0}\right)+\lambda_{1}\left(\alpha_{1}\|\beta {\|}_{1}+\left(1-\alpha_{1}\right)\|\beta {\|}_{2}^{2}/2\right)\right)$$

where *λ*_1_ sets the regularisation strength, *α*_1_ ∈ [0, 1] sets the fraction of the penalty assigned to the *ℓ*^1^-norm, and $\tilde{\beta }$ is a (*p* + 1) -dimensional vector that stacks the optima of *β* and *β*_0_. Then, in a second stage, we relaxed the coefficients using ridge regression restricted to the subset of predictors identified by the naïve elastic net:

(Equation 8) $$\beta^{*}={\text{argmin}}_{\beta ,\beta_{0}}\left(-\frac{1}{n}\ell \left(\beta \circ 1_{ℳ},\beta_{0}\right)+\lambda_{2}\|\beta {\|}_{2}^{2}\right)$$

where ∘ denotes the elementwise (Hadamard) product of two vectors, **1**_ℳ_ is one for the set of predictors ℳ⫅{1; …; *p*} assigned non-zero weights by the naïve elastic net regression and zero otherwise, *λ*_2_ sets the regularisation strength, and β* is a (*p* + 1) -dimensional vector that stacks the optima of *β* and *β*_0_. Note that β* is zero for all predictors outside of *M*. We finally used cross-validation to simultaneously optimize *α*_1_, *λ*_1_, and *λ*_2_ and estimate the model parameters, denoted $\left(\hat{\beta },{\hat{\beta }}_{0}\right)$. In practice, we used the glmnet package and *k* -fold cross-validation (typically with *k* = 10).

It is helpful to outline the details of this relaxed regularisation procedure with pseudocode:

for *a* ∈ {0:001; 0:25; 0:5; 0:75; 0:999}

    £_1_ : = glmnet *λ*_1_ penalty path for *α*_1_ = *a* and dataset *X; Y*

    for *l*_1_ ∈ {£_1_; …; £_10_} (10 values along penalty path)

        $X_{I_{1}}$: = predictor subset at *l*_1_

        £_2_ : = glmnet *λ*_2_ penalty path for *α*_2_ = 0 and dataset $X_{I_{1}}$, *Y*

        *l*_2_ : = largest penalty in £_2_ within 1 s.e. of minimal cross-validated deviance

        β*(*a; l*_1_; *l*_2_) : = glmnet model for *α*_2_ = 0; *λ*_2_ = *l*_2_ and dataset $X_{I_{1}}$, *Y*

        *D*(*a; l*_1_; *l*_2_) : = cross-validated deviance for β*(*a; l*_1_; *l*_2_)

    end

end

$\left(\alpha_{opt},\lambda_{1_{opt}},\lambda_{2_{opt}}\right)$ : = minimum of *D*(*a; l*_1_; *l*_2_) $\left(\hat{\beta },{\hat{\beta }}_{0}\right):=\beta^{*}\left(\alpha_{opt},\lambda_{1_{opt}},\lambda_{2_{opt}}\right)$

In words, we fit ridge regression models restricted to the subset of predictors that were selected at ten equally spaced positions along the elastic net solution path. The model with the greatest predictive power, as assessed by cross-validation, was selected.

##### Goodness-of-fit

Goodness-of-fit was quantified by the cross-validated fraction of Poisson deviance explained. If *ℓ_sat_* is the log-likelihood of the saturated model (with a free parameter per observation), *ℓ*_*model*_ is the cross-validated log-likelihood of the fitted model, and *ℓ*_0_ is the cross-validated log-likelihood of the null (*β*_0_ only) model, then the cross-validated deviances are

(Equation 9) $$D_{model}=2\cdot \left(\ell_{sat}-\ell_{model}\right)$$

(Equation 10) $$D_{null}=2\cdot \left(\ell_{sat}-\ell_{null}\right)$$

such that the cross-validated fraction of null deviance explained by the model is:

(Equation 11) $${ℛ}^{2}=1-\frac{D_{model}}{D_{null}}$$

##### Motor kinematic predictors

The predictors for regression modelling were derived from bout kinematics. Because we expected that some RSNs might modulate their activity specifically in response to leftwards- or rightwards-directed motor activity,^24^ we split lateralised motor features into pairs of positively-signed predictors representing rectified activity towards the left or right (Table S1). In addition, we included a motionerror predictor (the translation distance applied to each imaging frame) to capture variance attributable to any residual sample motion. All predictors were scaled to unit standard deviation.

We fit regression models directly in the basis of these motor kinematics (‘kin models’) and in the basis of their singular vectors (‘SVD models’), because we expected singular value decomposition to improve the consistency of the elastic net’s parameter selection by decorrelating the predictor matrix. In the former case, we denote the fit parameters as $\left({\hat{\beta }}_{kin},{\hat{\beta }}_{0,kin}\right)$. In the latter case, we applied SVD:

(Equation 12) $$X=U\Sigma V^{⊤}$$

to rewrite *X* as a product of an *n* × *n* orthogonal matrix (*U*), an *n* × *p* rectangular diagonal matrix (Σ), and a *p* × *p* orthogonal matrix (*V_T_*). Columns of the orthogonal matrix *V* define a *p* -dimensional basis, and we defined *X*_*svd*_ to be the predictor matrix in this singular vector basis

(Equation 13) $$X_{svd}=XV=U\Sigma$$

As expected, the SVD predictor matrix is decorrelated ($X_{svd}^{T}X_{svd}=\Sigma^{T}\Sigma$ is diagonal). We let $\left({\hat{\gamma }}_{svd},{\hat{\beta }}_{0,svd}\right)$ denote the model parameters fit in this basis, where the notation *γ* is used in place of *β* to emphasize that the model weights are not represented in the usual basis. To facilitate interpretation, model coefficients were transformed back to the basis of the motor kinematics:

(Equation 14) $${\hat{\beta }}_{svd}=V{\hat{\gamma }}_{svd}$$

##### Identification of functional archetypes

We reasoned that a principled way of identifying neurons whose activity was modulated in a similar way in relation to motor variables was to compare patterns of kinematic modulation across a large, common set of swim events. To this end, we pseudorandomly selected 5573 swim bouts from our dataset, with even sampling across swim bout types and fish. This defines a predictor matrix *X*_*g*_, where each row of *X*_*g*_ describes the kinematics of a single swim bout. Next, for every neuron, we predicted its kinematic modulation, $\hat{Z}$, and mean activity, *Ŷ*, as:

(Equation 15) $$\hat{Z}=X_{g}\hat{\beta };\;\hat{y}=e^{\overset{`}{Z}+{\hat{\beta }}_{0}}=e^{\hat{Z}}\cdot e^{\hat{\beta_{0}}}$$

where $\left(\hat{\beta },{\hat{\beta }}_{0}\right)$ denotes the best fitted encoding model (highest *R*^2^). Thus, $e^{\hat{Z}}$ modulates, in a kinematic specific manner, the baseline activity of the cell (defined by the model bias *β*_0_). Its log transform, $\hat{Z}$, facilitates balanced consideration of positive and negative modulation, and so we implemented a multi-stage clustering procedure to identify groups of neurons with similar $\hat{Z}$ vectors, as follows.

1. *Batch-clusters*. We first performed hierarchical agglomerative clustering of neurons whose best regression models had *R*^2^ > 0:3 (approximately the mean of the population distribution). Clustering was performed on $\hat{Z}$ vectors using a correlation distance metric and, for computation efficiency, we performed clustering using pseudorandom subsets of ~ 4000 neurons to generate a library of ‘batch-clusters’.
2. *Merging of batch-clusters*. Because batch-clusters were generated from subsets of the data, we next needed to combine similar batch-clusters. To do this, we generated a distance matrix where the distance between each pair of batch-clusters was the correlation distance between their centroids scaled by the median intra-cluster correlation distance of the more compact cluster. Using this distance matrix, we performed multiple iterations of hierarchical clustering, with the minimum number of clusters varying from 10 to 50. This enabled us to construct an evidence accumulation matrix, *EA*, quantifying the fraction of iterations in which each batch-cluster pair was assigned to the same cluster. Finally, 1 − *EA* defined a distance matrix on which we performed hierarchical clustering, cutting the dendrogram at the longest link. In this way, we merged the batch-clusters into 30 preliminary clusters.
3. *Cluster curation*. Clusters were trimmed by removing neurons located beyond the 70th percentile of the distribution of correlation distances to the cluster centroid. By manual inspection we then eliminated small and heterogeneous clusters, leaving eight clusters (comprising 71% of clustered cells).
4. *Functional archetype assignment*. Finally, for every neuron we evaluated the correlation distance between its $\hat{Z}$ vector and the eight cluster centroids. Neurons were assigned to the closest cluster below a threshold distance, which was set to twice the threshold imposed in the previous curation stage. The resulting 8 sets of cells are referred to as functional archetypes.

##### Uniform Manifold Approximation and Projection (UMAP)

For visualisation and interpretation, we used a MATLAB implementation^105^ of UMAP^106^ to project the matrix of $\hat{Z}$ vectors into a 2D space (run_umap, metric=correlation). Projected data points were then colour-coded according to functional archetype or anatomical cell label.

##### Kinematic tuning of functional modules

We evaluated kinematic tuning for each functional archetype using OASIS-inferred spiking activity and simultaneously recorded swim bout kinematics. For each kinematic feature, we defined 10 bins containing an approximately equal number of bouts and for each neuron we then evaluated the mean number of OASIS-inferred spikes at each bin. Tuning curves were generated by taking the mean across cells in each functional archetype.

##### Decoding swim types and motor kinematics

We trained linear decoders to predict swim bout label or motor kinematics from model-predicted neuronal activity. For each of ten iterations, an RSN population of 288 cells was pseudorandomly generated from our dataset, comprising the mean number of cells of each anatomical label as was observed across 67 animals. A sample of 700 swim bouts was also pseudorandomly selected, evenly drawn from the 14 bout type labels. A spiking process with Poisson-distributed error was simulated (MATLAB poissrnd) using model-predicted activity (*ŷ*, above) for these cells and swim events. For decoders based on functional archetypes or anatomical labels, spikes were averaged by taking the mean across cells assigned to each archetype or anatomical label, respectively. Linear LASSO decoders were trained using ten-fold cross-validation (glmnet, alpha=1, model=multinomial (bout type) or model=mgaussian (kinematics), mtype=grouped) to predict bout type label or the vector of kinematic regressor values.

### Quantification and Statistical Analysis

All statistical analyses were performed in MATLAB. Types of statistical test and *N* are reported in the text or figure legends. All tests were two-tailed and we report *p*-values without correction for multiple comparisons unless otherwise noted.

## Supplementary Material

Supplemental information can be found online at https://doi.org/10.1016/j.cub.2025.07.066.

Supplementary Material

## Data Availability

Processed data have been deposited at Mendeley Data as https://doi.org/10.17632/v8wd82hkg9.2 and are publicly available as of the date of publication. Raw data reported in this paper will be shared by the lead contact upon request but has not been deposited due to its large size. All original code has been deposited at Mendeley Data and is publicly available at https://doi.org/10.17632/v8wd82hkg9.2 as of the date of publication. Any additional information required to reanalyze the data reported in this paper is available from the lead contact upon request.

## Highlights

- Calcium imaging of >18,000 reticulospinal neurons during varied locomotor behaviors
- Modeling single-neuron activity reveals a low-dimensional functional organization
- Supraspinal control is modular, mediated by 8 functional archetypes
- Five archetypes control most locomotion, while 3 are specialized for hunting

## In brief

Lau et al. use calcium imaging and statistical modeling to comprehensively survey reticulospinal activity during diverse locomotor behaviors. They find that a small set of functional modules act combinatorially to generate descending commands, and unexpectedly, this control architecture is stratified according to behavioral context.
