# Supplementary material for: Supraspinal commands have a modular organization that is behavioral context specific
Source: Curr Biol. Author manuscript; Available in PMC 2025 Dec 20. (PMC7618495; doi:10.1016/j.cub.2025.07.066)
Supplement: Supplementary Material [file EMS211313-supplement-Supplementary_Material.zip › 1-s2.0-S0960982225010036-mmc1.pdf]

Current Biology, Volume *35*

Supplemental Information

Supraspinal commands have a modular organization that is behavioral context specific

Joanna Y.N. Lau, James E. Fitzgerald, and Isaac H. Bianco

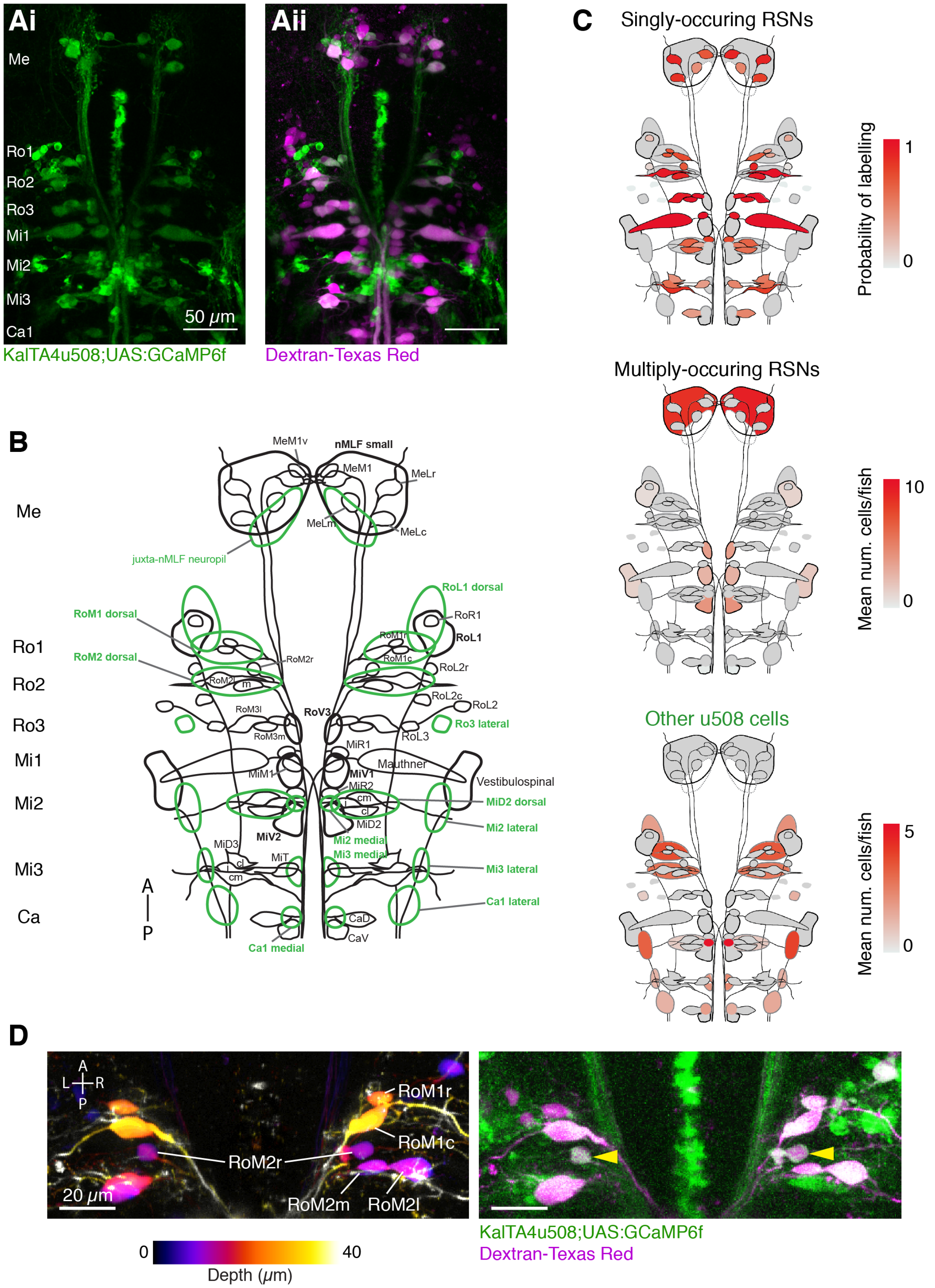

Ai
Me
Ro1
Ro2
Ro3
Mi1
Mi2
Mi3
Ca1
50 μm
KalTA4u508;UAS:GCaMP6f
Aii
Dextran-Texas Red
C
Singly-occuring RSNs
Probability of labelling
1
0
Multiply-occuring RSNs
Mean num. cells/fish
10
0
Other u508 cells
Mean num. cells/fish
5
0
B
Me
Ro1
Ro2
Ro3
Mi1
Mi2
Mi3
Ca
A
P
MeM1v
nMLF small
MeM1
MeLr
MeLm
MeLc
juxta-nMLF neuropil
RoL1 dorsal
RoR1
RoL1
RoM1 dorsal
RoM2 dorsal
RoM2r
RoM1r
RoM1c
RoL2r
RoL2c
RoL2
RoM3l
RoV3
Ro3 lateral
RoM3m
RoL3
MiR1
Mauthner
MiM1
MiV1
MiR2
Vestibulospinal
MiD2 dorsal
MiD2
Mi2 lateral
MiV2
MiD3
Mi2 medial
Mi3 medial
MiT
Mi3 lateral
Ca1 lateral
CaD
CaV
Ca1 medial
D
A
L
R
P
RoM1r
RoM1c
RoM2r
20 μm
RoM2m
RoM2l
0
40
Depth (μm)
KalTA4u508;UAS:GCaMP6f
Dextran-Texas Red

Figure S1: Neuronal labelling in u508:GCaMP6f transgenic, related to Figure 1.

(A) Example of spinal backfill labelling in u508:GCaMP6f transgenic animal. (i) GCaMP6f expression alone and (ii) with dextran-conjugated dye signal overlaid. (B) Anatomical labels used throughout this study. Black labels indicate canonical RSNs identified in spinal backfill studies, whereas those in green are additional u508 cell types. Labels on the left indicate levels of the segmentally organised reticular formation. Me, mesencephalon. Ro, rostral, Mi, middle and Ca, caudal regions of hindbrain correspond to rhombomeres 1–7. (C) Frequency of cell type labelling in u508:GCaMP6f. *Top:* Probability of labelling singly-occurring cell types. *Middle, bottom:* Mean number of cells per animal for multiply-occurring cell types. Note that some lateral RSNs (e.g. RoL2/3) are difficult to label by spinal backfill and so the probability of u508 expression is difficult to quantify. $N = 19$ animals. (D) RoM2r is a singly-occurring RSN in the Ro2 segment. *Left:* Backfill labelling; *Right:* Backfill overlaid with u508:GCaMP6f expression. Yellow arrowheads mark RoM2r somata.

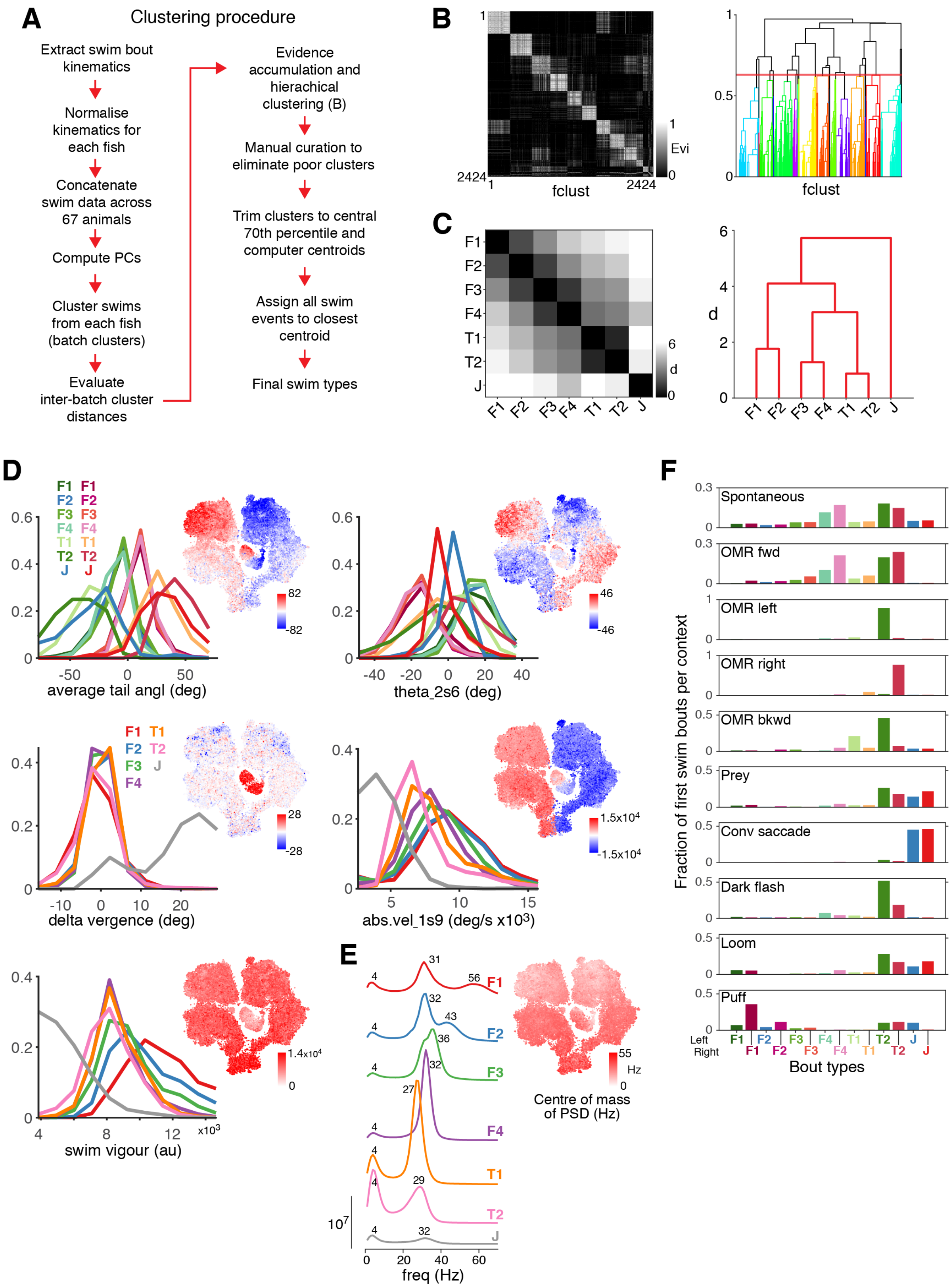

A
Clustering procedure
Extract swim bout kinematics
Normalise kinematics for each fish
Concatenate swim data across 67 animals
Compute PCs
Cluster swims from each fish (batch clusters)
Evaluate inter-batch cluster distances
Evidence accumulation and hierachical clustering (B)
Manual curation to eliminate poor clusters
Trim clusters to central 70th percentile and computer centroids
Assign all swim events to closest centroid
Final swim types
B
fclust
Evi
C
F1 F2 F3 F4 T1 T2 J
d
D
average tail angl (deg)
theta_2s6 (deg)
delta vergence (deg)
abs.vel_1s9 (deg/s x10^3)
swim vigour (au)
E
freq (Hz)
Centre of mass of PSD (Hz)
F
Spontaneous
OMR fwd
OMR left
OMR right
OMR bkwd
Prey
Conv saccade
Dark flash
Loom
Puff
Fraction of first swim bouts per context
Left
Right
Bout types

Figure S2: Swim bout types and clustering procedure, related to Figure 2.

(A) Multi-stage clustering procedure (see Methods for details). (B) *Left:* Evidence accumulation matrix quantifying the frequency with which 'batch cluster' pairs are assigned to the same cluster. *Right:* Dendrogram derived from evidence accumulation. Red line marks where dendrogram was cut to define clusters. (C) Distance matrix (left) and dendrogram (right) quantifying pairwise similarity between bout types. Distances were measured as correlation distance between cluster centroids scaled by within-cluster standard deviation. Left- and right-lateralised swims pooled within each type. (D) Histograms and t-SNE maps showing distributions of additional kinematics either for all 14 bout labels (top row) or the seven bout types (remaining plots; left and right swims pooled). *average tail angle* (morphAI2), mean tail angle during first 120 ms of swim bout; *theta_2s6*, bend angle for segment 6 during half-beat 2; *vel_1s9*, peak angular velocity at segment 9 during half-beat 1; *delta vergence* (Vdelta), change in ocular vergence; *swim vigour* (vigmax), maximum swim vigour during bout. (E) Mean power spectral density for each bout type. Numbers indicate position (Hz) of the corresponding peaks. Inset shows centre of mass of PSD across t-SNE embedding space. (F) Deployment of different bout types in different contexts. Plots show probability that first swim in each context belongs to a given type.

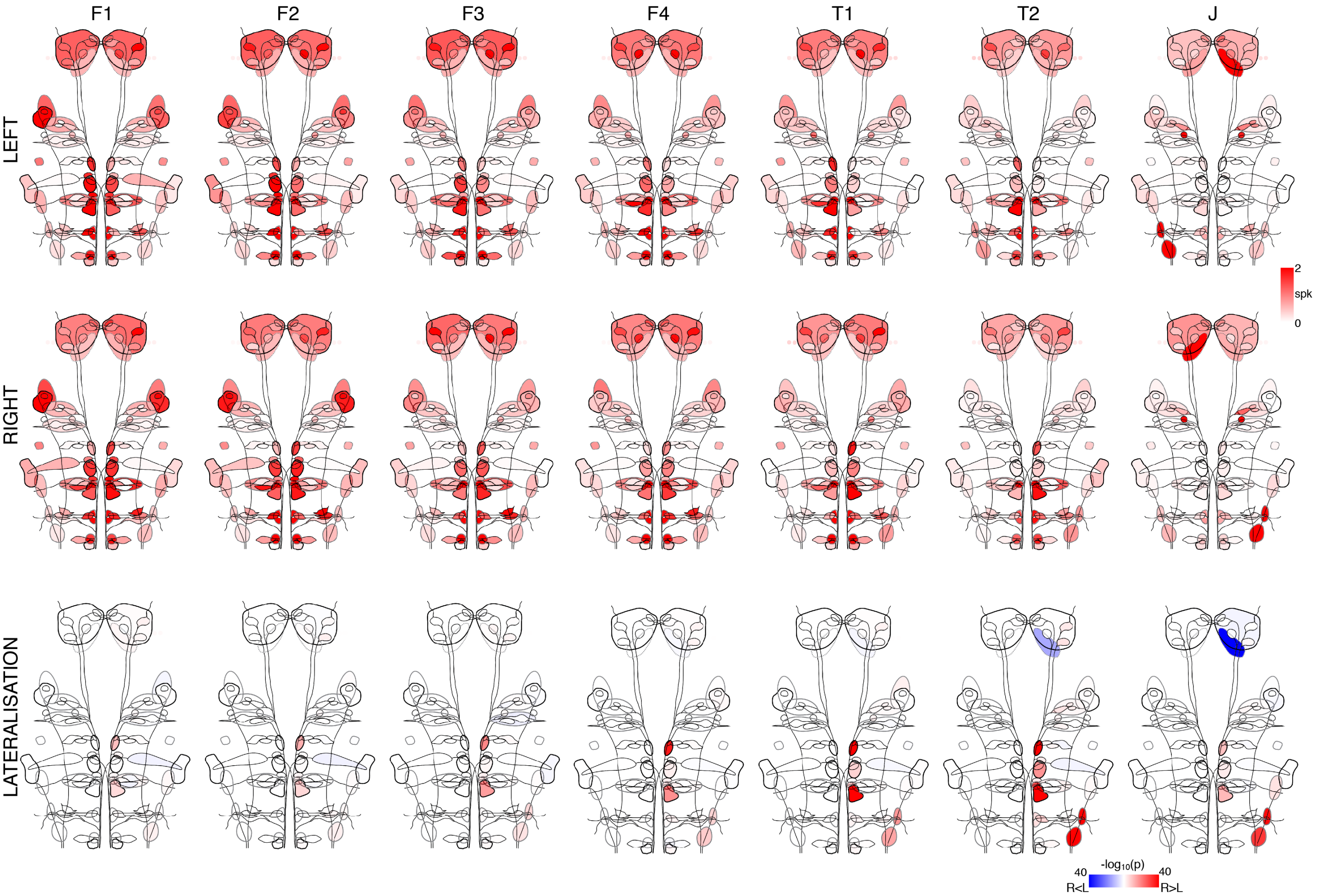

Figure S3: Reticulospinal activity and lateralisation, related to Figure 3.

Top, Middle: Activity maps, as per [Figure 3], for left- and right-lateralised swim bouts of each of the seven types. *Bottom:* Right–left asymmetry of RSN activity. For each (right-lateralised) bout type, we compared spike count distributions between corresponding neurons on right versus left side of the brain (Mann-Whitney tests).

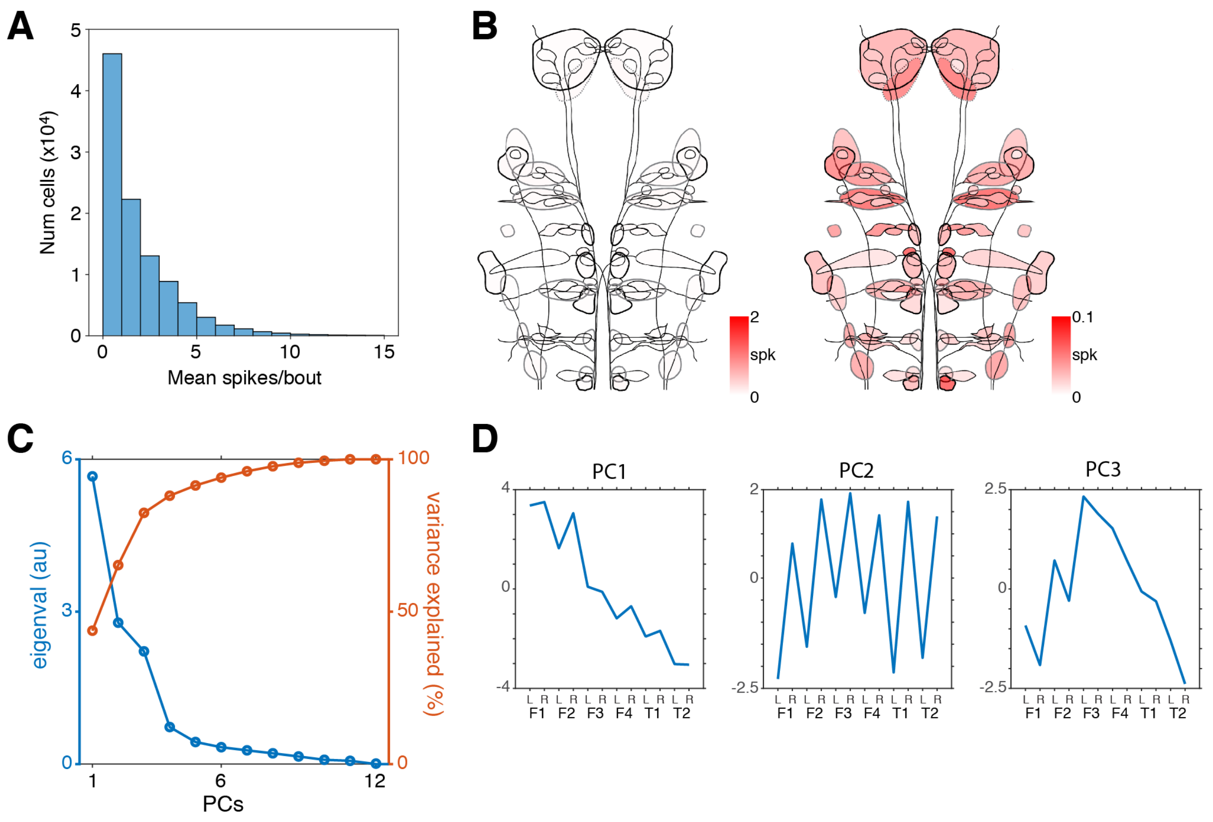

Figure S4: RSN recruitment - further details, related to Figure 3.

(A) Distribution of mean OASIS-inferred spikes per swim bout type. (B) Activity map for 'null' bouts, randomly sampled from periods when fish were not swimming. *Left:* Same colour scale as main figure. *Right:* Adjusted scale. (C) Eigenvalues for PCs (same analysis as [Figure 3C,D]). (D) Variation in PC1–3 across the kinematic sequence (PC1,2 data as per [Figure 3D]).

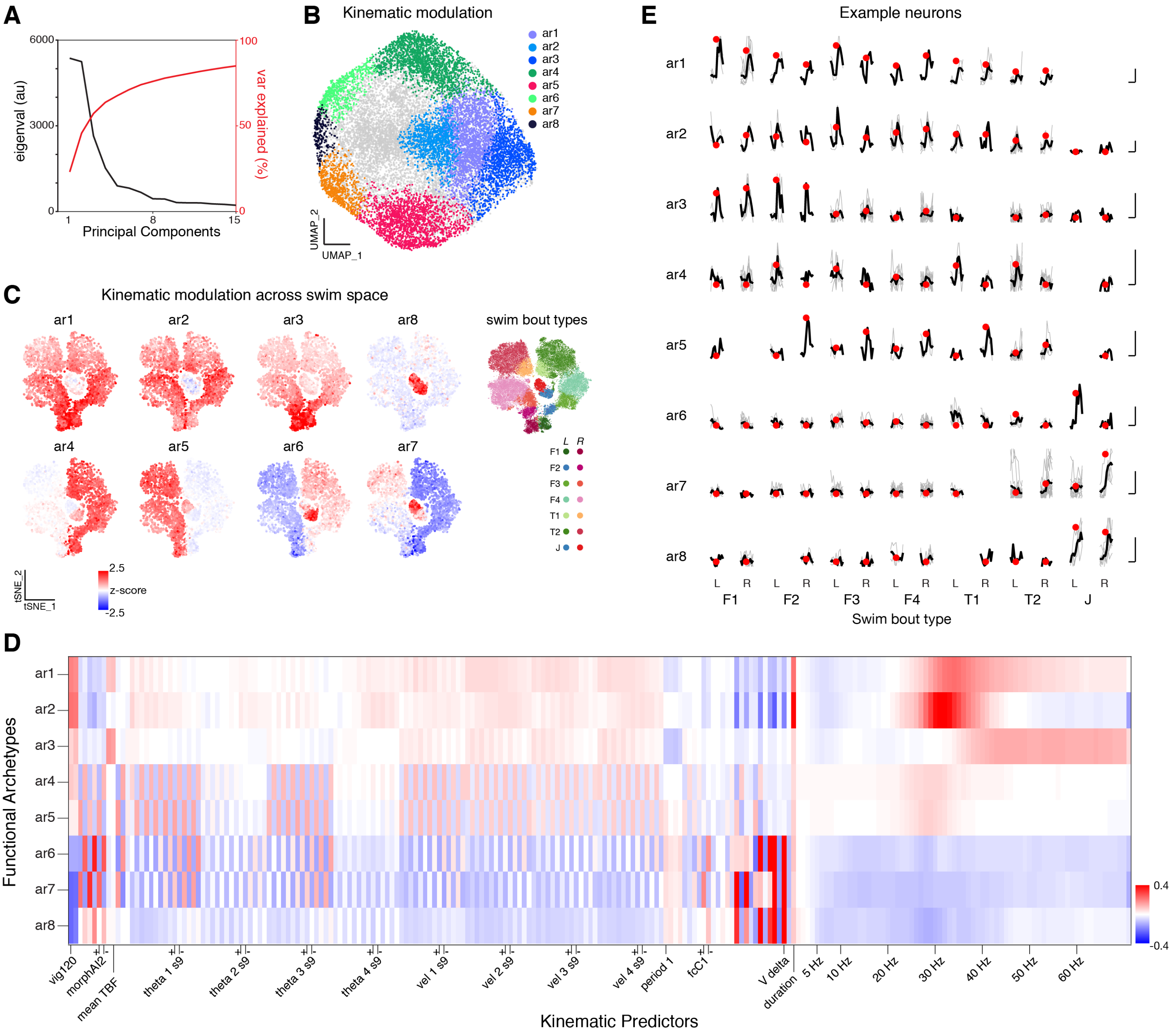

Figure S5: Functional archetypes - further details, related to Figure 5.

(A) Eigenvalues and cumulative variance explained by principal components of the kinematic modulation ($X\beta$) matrix. (B) UMAP embedding of kinematic modulation vectors. Each point represents one cell and those assigned to functional archetypes are coloured as per legend. (C) t-SNE embedding of swim bouts, colour-coded by mean kinematic modulation across cells assigned to each functional archetype. Rightmost panel is coded by bout type (reproduced from [Figure 2]). (D) Mean ENET models, as per [Figure 5E], with more detailed annotation of kinematic predictors. For directional kinematics, pairs of unsigned predictors represent leftwards ($-$) and rightwards ($+$) motor output. See [Table S1] for full list. (E) Example neurons from functional archetypes. Each row shows data from one cell, sampled from the first 30th percentile of distances to the archetype centroid. Calcium data for individual swims is shown in grey with the mean in black. Red dots show the mean number of OASIS-inferred spikes and blanks indicate the animal did not produce the corresponding bout type during imaging of the cell. Scale bars: 1 s and 5 zF/spikes.

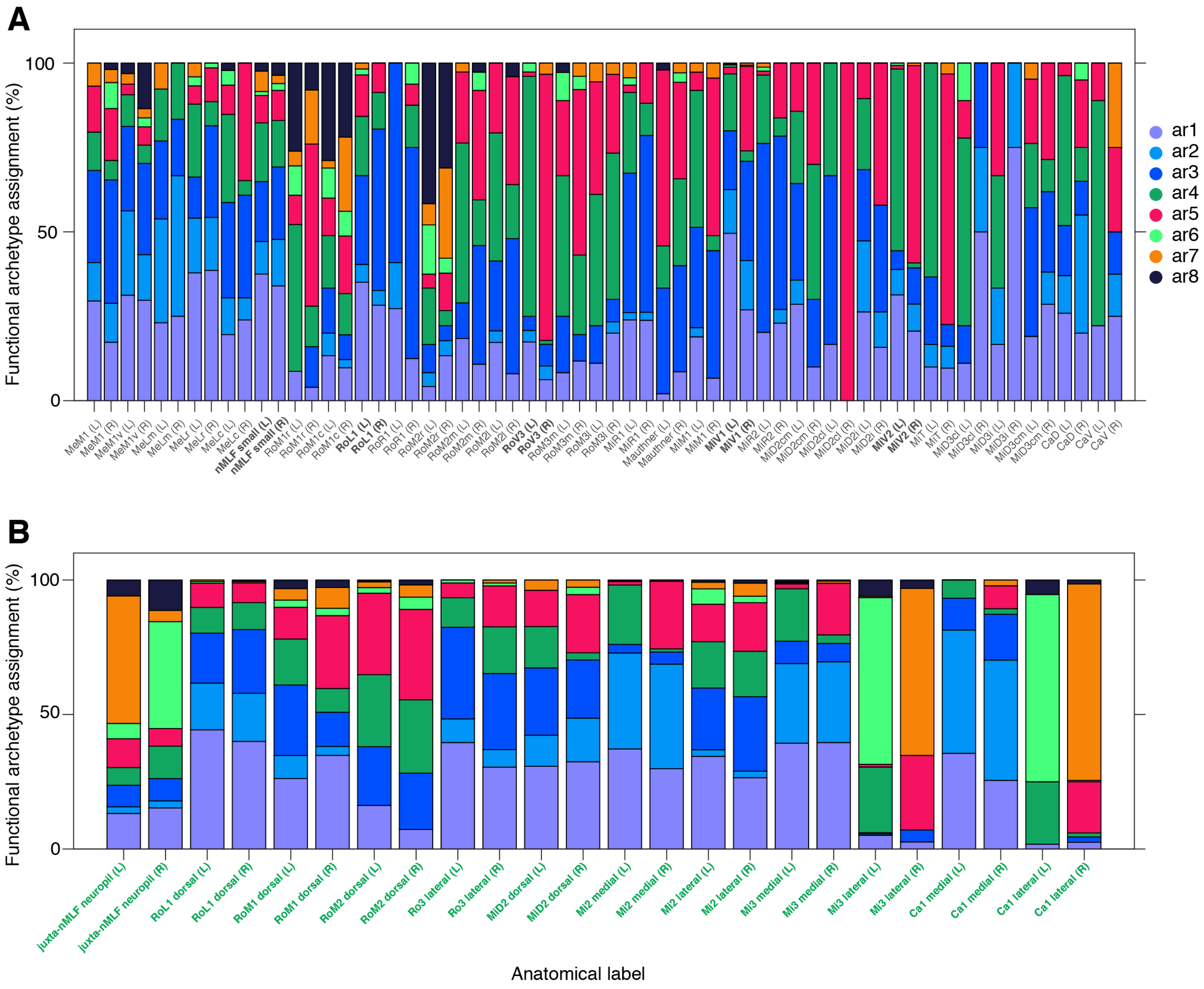

Figure S6: Functional archetype assignment for anatomically defined cell types, related to Figure 5.

Percentage of cells belonging to each anatomical label that were assigned to each functional archetype. (A) Canonical RSNs. (B) Additional cell labels identified in the u508:GCaMP6f line. Labels in bold indicate multiply-occurring cell types.

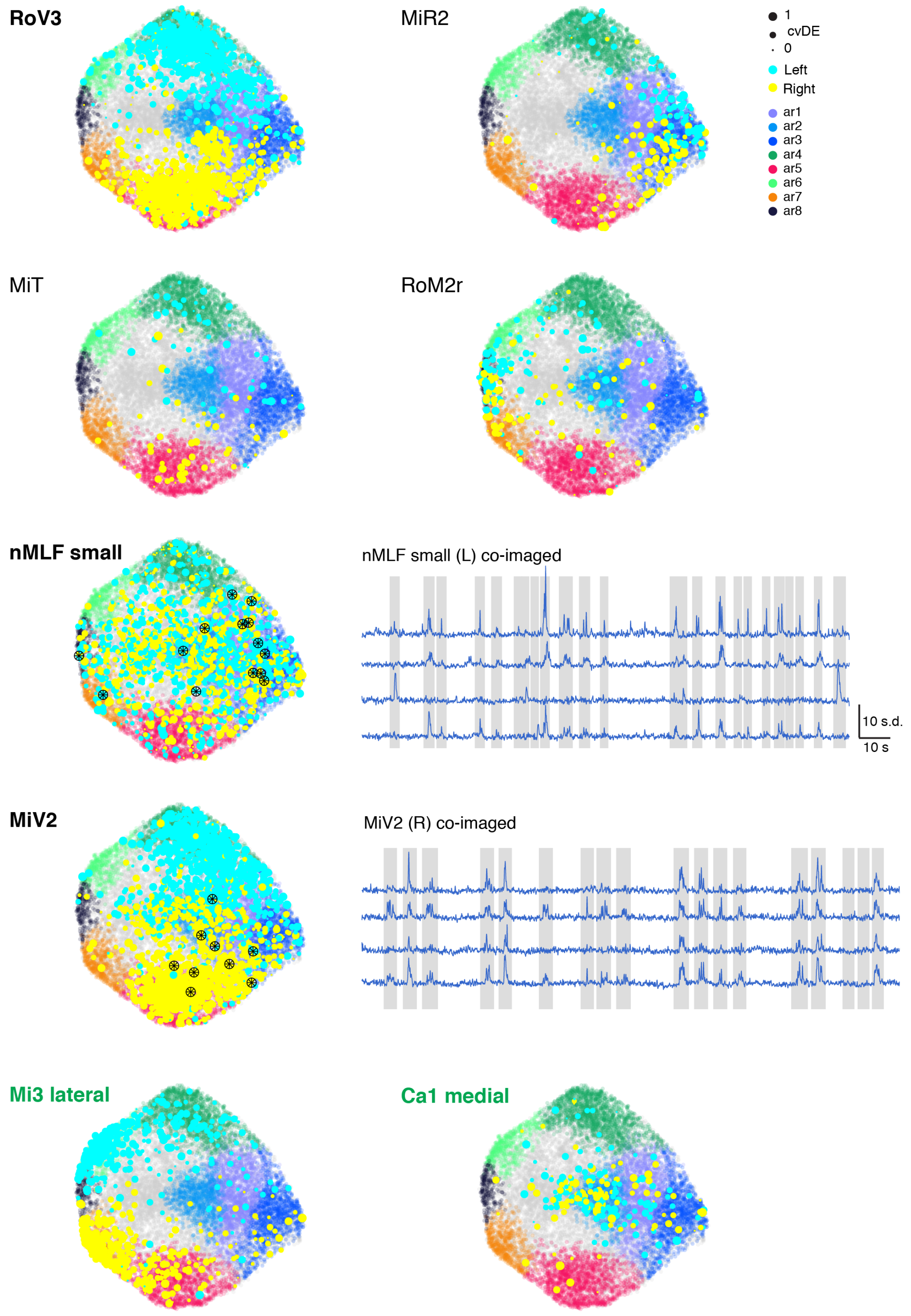

Figure S7: Anatomical cell types show diverse functional properties, related to Figure 5.

Each panel shows cells belonging to a specific anatomical label (left-sided cells in cyan, right-sided in yellow, symbol size represents $\mathcal{R}^2$ for best regression model) overlaid on UMAP embedding of all kinematic modulation vectors (as per [Figure S5B]). For nMLF small cells and MiV2 neurons, a subset of cells that were simultaneously imaged in one animal are indicated by ⊛ and calcium fluorescence data is shown for four cells from each subset (grey shading indicates swim bouts).

Name	Brief Description	Units	Lateralised	Index Kinematic	Index Regressor(s)
vig120	Sum vigour during first 120 ms	au	0	1	1
vigmax	Max vigour	au	0	2	2
intcum60ms	Integral of tail angl during first 60 ms	deg	1	3	3, 4
morphAI	Bout asymmetry measure ([-1, +1])	none	1	4	5, 6
morphAI2	Mean bend angl during first 120 ms	deg	1	5	7, 8
max_TBF	Max tail beat frequency	Hz	0	6	9
mean_TBF	Mean tail beat frequency	Hz	0	7	10
max_angl	max (absolute) tail bend angl	deg	1	8	11, 12
max_vel	max (absolute) tail angular velocity	deg/s	1	9	13, 14
theta_1_s5-11	bend angl at halfbeat 1 for segments 5 to 11	deg	1	10–16	15–28
theta_2_s5-11	bend angl at halfbeat 2 for segments 5 to 11	deg	1	17–23	29–42
theta_3_s5-11	bend angl at halfbeat 3 for segments 5 to 11	deg	1	24–30	43–56
theta_4_s5-11	bend angl at halfbeat 4 for segments 5 to 11	deg	1	31–37	57–70
vel_1_s5-11	peak angl velocity at halfbeat 1 for segments 5 to 11	deg/s	1	38–44	71–84
vel_2_s5-11	peak angl velocity at halfbeat 2 for segments 5 to 11	deg/s	1	45–51	85–98
vel_3_s5-11	peak angl velocity at halfbeat 3 for segments 5 to 11	deg/s	1	52–58	99–112
vel_4_s5-11	peak angl velocity at halfbeat 4 for segments 5 to 11	deg/s	1	59–65	113–126
period_1–4	duration of halfbeat 1 to 4	s	0	66–69	127–130
fcR1	fraction curvature for halfbeat 1 in rostral tail	none	1	70	131, 132
fcM1	fraction curvature for halfbeat 1 in middle tail	none	1	71	133, 134
fcC1	fraction curvature for halfbeat 1 in caudal tail	none	1	72	135, 136
ratio_tp1	ratio of bend angl for second vs first halfbeat	none	1	73	137, 138
ratio_period2v1	ratio of duration of halfbeat 2 vs 1	none	0	74	139
ratio_theta2v1	ratio of bend angl for halfbeat 3 vs 1	none	1	75	140, 141
Lpost	Post-bout position of left eye	deg	1	76	142, 143
Ldelta	Change in left eye position	deg	1	77	144, 145
Rpost	Post-bout position of right eye	deg	1	78	146, 147
Rdelta	Change in right eye position	deg	1	79	148, 149
Vpost	Post-bout ocular vergence	deg	1	80	150, 151
Vdelta	Change in ocular vergence	deg	1	81	152, 153
duration	Total duration of swim bout	s	0	82	154
fourierpsd_1-70	Fourier psd 1 to 70 Hz	au	0	83–152	155–224
motionerror	frame-wise motion correction	pixels	0	n.a.	225

Table S1: Kinematics and GLM regressors, related to Figures 2, 4 and 5.

See Methods for description of how each kinematic was computed. Kinematics that describe movement relative to the left–right axis are indicated by the **Lateralised** column. These were converted to pairs of unsigned regressors, with consecutive indices, describing rightwards (+) and leftwards (-) motion. e.g. theta_1_s9 was converted to theta_1_s9+ (index 23) and theta_1_s9- (index 24) to describe rightwards and leftwards bends, respectively. au: arbitrary units.
